# Reactivity of aminophenols in forming nitrogen-containing brown carbon from iron-catalyzed reactions

**DOI:** 10.1038/s42004-022-00732-1

**Published:** 2022-09-19

**Authors:** Hind A. Al-Abadleh, Fatemeh Motaghedi, Wisam Mohammed, Md Sohel Rana, Kotiba A. Malek, Dewansh Rastogi, Akua A. Asa-Awuku, Marcelo I. Guzman

**Affiliations:** 1 Department of Chemistry and Biochemistry, Wilfrid Laurier University, Waterloo, ON N2L 3C5 Canada; 2 Department of Chemistry, University of Kentucky, Kentucky, 40506 USA; 3 Department of Chemical and Biomolecular Engineering, University of Maryland, College Park, MD 20742 USA

**Keywords:** Atmospheric chemistry, Characterization and analytical techniques

## Abstract

Nitrogen-containing organic carbon (NOC) in atmospheric particles is an important class of brown carbon (BrC). Redox active NOC like aminophenols received little attention in their ability to form BrC. Here we show that iron can catalyze dark oxidative oligomerization of *o*- and *p*-aminophenols under simulated aerosol and cloud conditions (pH 1–7, and ionic strength 0.01–1 M). Homogeneous aqueous phase reactions were conducted using soluble Fe(III), where particle growth/agglomeration were monitored using dynamic light scattering. Mass yield experiments of insoluble soot-like dark brown to black particles were as high as 40%. Hygroscopicity growth factors (κ) of these insoluble products under sub- and super-saturated conditions ranged from 0.4–0.6, higher than that of levoglucosan, a prominent proxy for biomass burning organic aerosol (BBOA). Soluble products analyzed using chromatography and mass spectrometry revealed the formation of ring coupling products of *o*- and *p*-aminophenols and their primary oxidation products. Heterogeneous reactions of aminophenol were also conducted using Arizona Test Dust (AZTD) under simulated aging conditions, and showed clear changes to optical properties, morphology, mixing state, and chemical composition. These results highlight the important role of iron redox chemistry in BrC formation under atmospherically relevant conditions.

## Abstract (web summary)

Iron is the most abundant redox active transition metal in mineral dust, but its role in nitrogen-containing organic carbon formation remains largely unexplored. Here, the authors show that Fe(III) catalyzes the dark oxidative oligomerization of *o*- and *p*-aminophenols under simulated aerosol and cloud conditions.

## Introduction

Quantifying the contribution of atmospheric brown carbon (BrC) to climate forcing and aerosol-cloud interactions remains a large source of uncertainty in climate models due to their chemical complexity and variable sources^1–4^. Atmospheric BrC refers to the class of organic compounds that efficiently absorb solar and terrestrial radiation. The majority of BrC originates from biomass burning events and their cloud condensation nuclei activity and hygroscopicity parameter, κ, ranges from 0.1 to 0.4, which slightly increases upon particle photochemical or oxidative aging^5^. Nitrogen-containing organic carbon (NOC) in atmospheric particles are increasing in importance as a class of BrC^6–8^, which originates from primary and secondary sources^9,10^. Primary emission sources of NOC particles were estimated to be 27.4 Tg yr^−1^ from biogenic, soil, ocean, and anthropogenic (that includes biomass burning) sources^9^. Molecular characterization and microscopy analysis of biomass burning organic aerosols (BBOA) including tar balls showed uniform distribution of nonvolatile NOC^11^, with only 14 ± 3% of the N-bearing compounds occur as ammonium sulfate^12^. Recent advances in analytical tools allowed for direct measurements of inorganic (ammonium, nitrite, and nitrate) and organic nitrogen in ambient aerosol samples as opposed to the difference method between total nitrogen and inorganic nitrogen^13^.

There are two major NOC classes in BrC that reflect the oxidation state of nitrogen: oxidized NOC that include nitrocatechols and nitrophenols, and reduced NOC that includes amines, imines and N-heterocycles (e.g., pyridines and pyrazines)^9,14^. In general, nitro-aromatic compounds are characterized as chemical tracers from biomass burning events along with levoglucosan, forming during pyrolysis of plant biopolymers^15–17^. Atmospheric amines include aliphatic and aromatic compounds^18,19^. Low molecular weight with 1–6 carbons aliphatic amines are the most abundant in the atmosphere with global emission fluxes dominated by trimethylamine (TMA) and ethanolamine from animal husbandry, marine and biomass burning sources^18^. Glycine was found to be the most abundant amino acid in atmospheric aerosols from the decay of biological matter^18,20,21^. The reactivity and hydration properties of some aliphatic amines were explored for their ability to form secondary organic aerosol (SOA)^22,23^ and act as cloud condensation nuclei (CCN)^21,24,25^. Aromatic amines in atmospheric particles on the other hand are dominated by phenylamine (i.e., aniline), *p*-aminophenol and phenylamine with alkyl substituents^26^. While the global fluxes of these molecules have not been reported, their concentrations in the gas phase and ultrafine particulate matter are source-dependent mostly from industrial emissions^18,26,27^. Multiphase redox chemistry could also lead to the formation of aromatic amines from the reduction of nitrobenzenes^28^ and nitrophenols^29,30^, which warrants further investigation under cloud and aerosol conditions. Supplementary Scheme 1 shows the structure and pK_a_ values of some of the abovementioned compounds with phenol and catechol as reference compounds. As summarized by Yu et al.^13^, the concentration of water-soluble organic nitrogen accounts for 10–30% of water-soluble total nitrogen and ranges from 1 to several hundred nmol N m^−3^ in remote oceanic to polluted urban areas, respectively.

Secondary sources of NOC that contribute to BrC constitute an active area of research^7^. For example, particle phase formation pathways between ammonia and primary amines and carbonyl groups lead to BrC formation^7^. Efficient formation of NOC was observed from ammonia gas phase uptake on newly formed secondary organic aerosol (SOA) from the oxidation of *α*-pinene and *m*-xylene^31^, and evaporation of glyoxal-ammonium sulfate droplets^32^. The interaction of ammonium with oxidized organics in single particles during summer and fall seasons at an urban location (Guangzhou, China) was found to explain 50% of NOC formation in processes facilitated by NO_x_ and relative humidity^33^. The reactive uptake of glyoxal into ammonium-containing salts or methylaminium-containing salts was also found to produce BrC at different RHs^34,35^. Direct photosensitized oxidation of vanillin, a phenolic carbonyl, in the presence of ammonium nitrate was also found to produced aqueous SOA^36^. Reactions with nitrogen dioxide (NO_2_) and nitrate radical (NO_3_) led to nitration of polycyclic aromatic hydrocarbons yielding nitrophenols, nitrocatechols^37–40^, nitration of isoprene-derived epoxides during SOA formation^41^, and nitro-heterocyclic compounds such as nitropyrrole^42^.

The role of transition metals in NOC formation and contribution to BrC remains largely unexplored^43,44^. Iron-containing atmospheric particles are ubiquitous in the atmosphere^45^ from natural sources such as in mineral dust^46^ and anthropogenic combustion including coal burning^47,48^, biomass burning^49,50^, and brake wear^51^. As recently highlighted in references^44,52^, there are realistic scenarios in which phenolic compounds and NOC from biomass burning and industrial sources encounter iron-containing particles through gas-particle partitioning or particle mixing. This is because emissions from these sources containing semivolatile gases and particles are often spread by wind, which also lifts crustal particles off the ground. Long-range transport of mineral dust^53^ and BBOA^54^ by wind contribute to atmospheric aging and growth of particles partly due to partitioning of organic vapors into Fe-containing particles and reactions with acids. Other pathways that lead to dust aging and particle growth remain unexplored. One potentially important pathway is the one catalyzed by iron since aging processes increase the solubility of iron which can reach millimolar levels in aerosol liquid water as reported by Gen et al.^55^.

We recently reported the formation of soluble NOC from dark reactions between Fe(III) and catechol that formed insoluble black polycatechol particles and colored water-soluble oligomers under conditions characteristic of viscous multicomponent acidic aerosol systems containing ammonium^56^. These studies suggested that the catalytic power of the catechol/*o*-quinone redox pair^57–59^ together with the complex equilibria for Fe(III) and Fe(II) ions with catechol and dicarboxylic acids should play a key role in the formation of secondary BrC. The compounds shown in Supplementary Scheme 1 contain functional groups (Ar–OH, –NH_2_, –C(O)OH) capable of complexing iron. The arylamines are electron-rich and redox active and can undergo enzymatic and electrochemical oxidation leading to oligomerization and polymerization^60–64^. Supplementary Table 1 lists the oxidation potential and major products of phenol, catechol and selected aromatic amines from electrochemical studies under acidic conditions. In systems containing Fe(III), the other half of the redox couple that corresponds to the reduction reactions are listed in Supplementary Table 2. For a system containing catechol/Fe(III) at pH 3, a net positive potential is obtained indicating spontaneous reaction under acidic conditions. Using the same approach, the redox reactions between phenol or aniline and Fe(III) are non-spontaneous under acidic conditions, whereas they are between *o*- and *p*-aminophenol (*o*AP and *p*AP). Metal-free oxidation of *o*AP and *p*AP results in the formation of polymeric products referred to as poly(*o*-aminophenol) and poly(*p*-aminophenol), which are desired for their corrosion inhibition properties^63^. The oxidation potential for glycine and other aliphatic amines is higher than 0.7 V at pH 1, hence, the redox reaction with Fe(III) would be non-spontaneous^65–67^.

The objective of this investigation is to explore the role of iron-catalyzed reactions in forming BrC from aromatic amines under conditions that mimic dark processing in cloud water, in the absence and presence of ammonium sulfate. Our studies here show the efficient and rapid formation of dark and insoluble NOC oligomers in addition to soluble products. The cloud condensation nucleation (CCN) efficiency of these non-combustion particles was also investigated and shown to be higher than levoglucosan. The iron content in Arizona Test Dust (AZTD) was also found to catalyze reactions with aminophenols over atmospherically relevant timescales that simulate long-range dust transport^68^ with impacts on their morphology and optical properties.

## Results

### Characterization of BrC from aminophenols

**Fig. 1:**
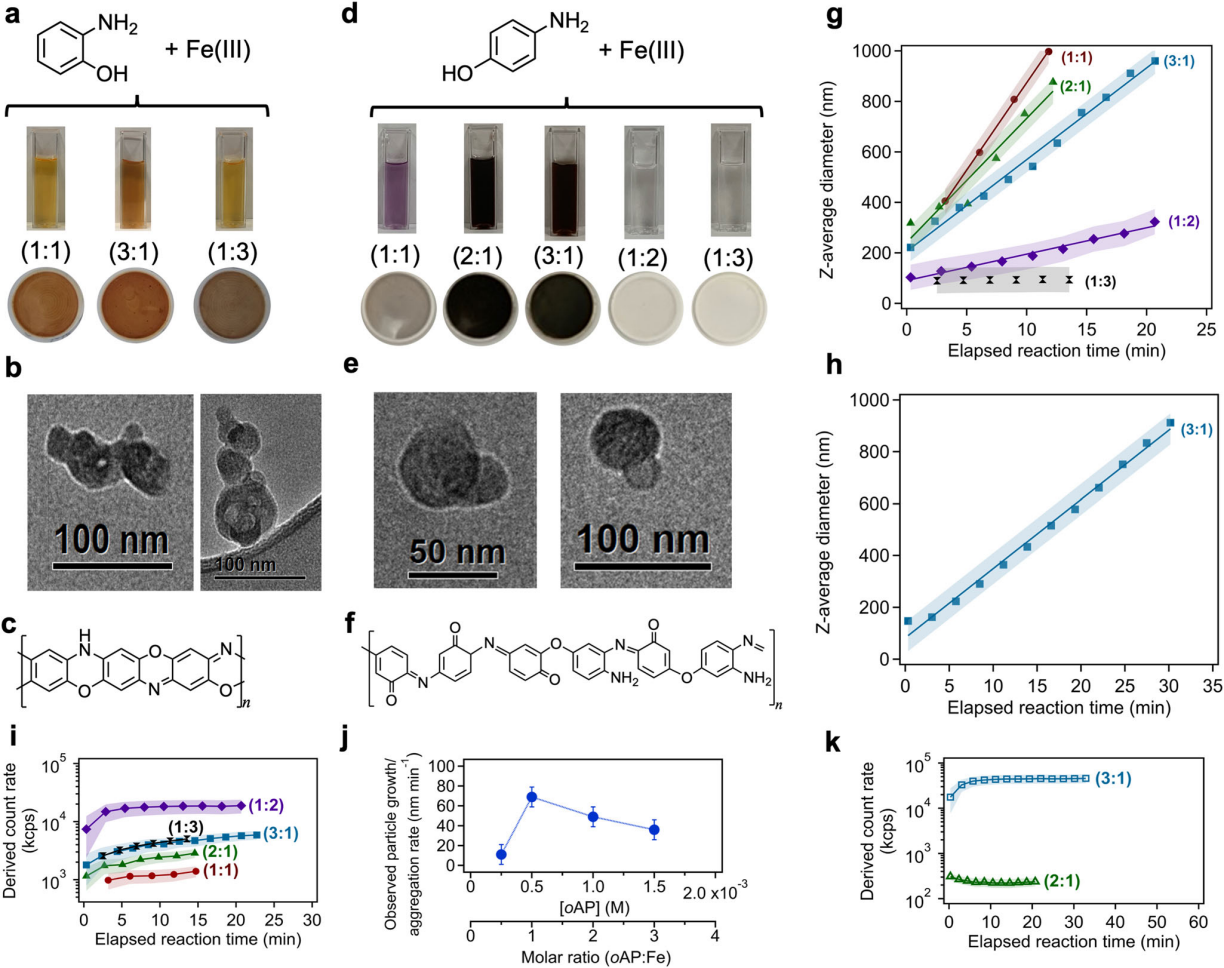
Qualitative and quantitative assessment of insoluble particle formation from the reaction of aminophenols with Fe(III). Summary of results for the dark aqueous phase reaction of *ortho*-aminophenol (*o*AP) (**a**–**c**, **g**, **i**, **j**) and *para*-aminophenol (*p*AP) (**d**–**f**, **h**, **k**) with Fe(III), as a function of the organic to Fe molar ratio. **a**, **d** Digital photographs of solutions after 40 min reaction (pH 3–4.5) in 0.01 M KCl background solution following DLS measurements, and dry filters after allowing the reaction to continue overnight (20 h). For **a**, [Fe(III)]_0_ = 0.5 mM and for **d**, [Fe(III)]_0_ = 2 mM. Similar photos for reaction products in 1 M (NH_4_)SO_4_ background solutions are shown in Supplementary Figure 1. **b**, **e** High resolution TEM images of the insoluble particles from the reactions with in **a** and **d**, respectively. Elemental analysis using energy dispersive x-ray spectroscopy (EDXS) is shown in Supplementary Table 4. **c**, **f** Suggested structures of poly(*o*-aminophenol) and poly(*p*-aminophenol) per reference (66). ATR-FTIR spectra and TGA curves are shown in Supplementary Figures 2 and 3, respectively. **g**, **h** DLS measurements of the average particle size of particles as a function of time (shaded area represents ±1 σ = 30%). Lines through the data correspond to linear least squares fitting. **i** Derived count rate, proportional to the scattering signal, from the particles forming in solution. **j** Observed rate of particle growth or aggregation as a function of [*o*AP] and molar ratio of *o*AP:Fe from the DLS data with error bars representing ±1σ. **k** Derived count rate, proportional to the scattering signal, from the particles forming in solution for the 3:1 and 2:1 *p*AP:Fe molar ratios. The derived count rate values for the 2:1 align with those for 2 mM FeCl_3_ solution (no organics, used as a reference solution), and hence, no size data is shown for this ratio.

The reaction of soluble Fe(III) with *o*AP and *p*AP was investigated as a function of the organic:Fe molar ratio under acidic conditions and two different background solutions, 0.01 M KCl (Fig. 1) and 1 M (NH_4_)SO_4_ (Supplementary Figure 1). For qualitative assessment of reaction progress, Fig. 1a and d show digital photographs for the reaction vials after 40 min reaction, which were filtered after overnight reaction time (~20 h). The photographs of the filters with the insoluble products are also shown. The organic concentrations were lower than 6 mM depending on the initial Fe(III) concentration and the desired organic to Fe(III) ratio. Detailed calculations on the atmospheric relevance of dissolved Fe(III) and aminophenol concentration are provided in the Supplementary Information document.

The mass yield of the insoluble products relative to the initial mass of the organic precursor ranged from 13 to 38% depending on pH and reactants molar ratio (Supplementary Table 3). Over 2 h, the qualitative dependence of particle density on pH is more obvious for *p*AP than *o*AP. According to Supplementary Table 1, the mechanism of electrochemical oxidation of *o*AP involves dimerization, whereas that of *p*AP involves hydrolysis of quinone imine to form benzoquinone. Increasing the pH increases the dimerization reaction rate (and hence oxidation potential) and decreases the hydrolysis reaction rate^69^. Supplementary Table 2 shows that decreasing the pH increases the reduction potential of iron species in solution^70^. Therefore, it is likely that redox reactions between the aminophenols/iron species pair become less thermodynamically favorable and the kinetics of the redox steps become slower with decreasing pH. Supplementary Scheme 2 shows the proposed formation of intermediate species following electron transfer to Fe(III) that leads to oligomerization/polymerization.

Fig. 1b,e show representative TEM-EDXS images of the colored solid products. These images show conglomerates of nanometer size soot-like particles containing below 1 wt% Fe and 0.4 wt% chlorine from using FeCl_3_ (Supplementary Table 4). Since the *o*AP reacted with Fe(III) over a wide range of molar ratios, the colored solid products were further analyzed using thermogravimetric analysis (TGA) to examine their thermal properties relative to that of poly(*o*-aminophenol) and poly(*p*-aminophenol) reported in the literature^63^. Supplementary Figure 2 highlights the thermal stability of the starting organic precursors *o*AP and the solid products from its dark aqueous phase reaction with Fe(III). *o*AP undergoes a one-step thermal degradation process that starts at 120 °C and ends around 200 °C during which the sample lost nearly 91% of its initial mass. However, the thermogram for the solid reaction product shows that the sample undergoes a three-stage decomposition pattern: ~267 °C with about 12% mass loss corresponding to the evaporation of water molecules and volatile compounds, ~321 °C with about 70% mass loss due to the loss of the oligomeric/polymeric part of the samples, and ~450 °C due to the thermal conversion of the iron content to *α*-Fe_2_O_3_, the most stable form of iron oxides at high temperature^71^, resulting in about 7% mass residue. The digital photos of the trays containing the samples show the dark red color of the residual material at the end of the TGA analysis. Supplementary Table 5 lists the %Fe and effective molar weight of color reaction products calculated from the residual mass percent of *α*-Fe_2_O_3_ (*x%*) to be 4.8% and 1159 g mol^−1^, respectively. The thermogram in Supplementary Figure 2 looks different than that reported for poly(*o*-aminophenol) suggesting structural differences between the two materials analyzed, which is expected given the differences in the experimental conditions that led to the polymerization of the aminophenols^63^. The average molecular weight determined for both poly(*o*-aminophenol) and poly(*p*-aminophenol) using gel permeation chromatography was 297,000 g mol^−1^ ^63^. Hence, the lower effective molar weight calculated above from our experiments suggests oligomers as the main products under our experimental conditions.

Moreover, Supplementary Figure 3 shows the ATR-FTIR absorbance spectra of dry thin films of the solid products from the reaction of Fe(III) with *o*AP and *p*AP with the assignment of observed vibrational modes. There are similarities between the spectra in Supplementary Figure 3 and those reported for poly(*o*-aminophenol)^63,64^ and poly(*p*-aminophenol)^63^ whose suggested structures are shown in Fig. 1c and f. However, the aromatic vibrations in Supplementary Figure 3 suggest different distributions of quinoid and benzenoid rings and the presence of amine and imine units. Mass spectra collected using MALDI in the negative mode of matrix-free solid thin films of these reaction products are shown in Supplementary Figure 4 for the mass range 210.5–213.5 of the dimers region. Clear mass features at [M-H]^−^ = 212.1 and 213.06 were observed and assigned to the dimer from the reaction of Fe(III) with *o*AP (*M*_wt_ = 109 g mol^−1^). These mass features were not observed using *p*AP as the organic precursor. The mass spectrum of the reactant FeCl_3_ is also shown as a reference. The most intense mass features were observed at the same locations for the thin films from the reaction of FeBr_3_ with *o*AP confirming their assignment to organic species. The mass ranges corresponding to the trimers and tetramers contained much less intense features or overlapping cluster features assigned to residual iron chloride and iron hydroxide species^72^. In summary, the characterization of the colored solid products suggests that the reaction conditions produced oligomers of *o*AP and *p*AP that are not as polymeric as those produced from metal-free polymerization reactions^63,64^.

Particle growth/agglomeration rate of *o*AP and *p*AP with Fe(III) was monitored in situ using DLS as shown in Fig. 1g, h. Figure 1i, k shows the derived count rate related to the scattering intensity confirming the presence of suspended particles in solution for values greater than 1000 kcps. Using *o*AP as the organic reactant (Fig. 1g), average particle size reached 1 μm in less than 30 min for the 1:1, 2:1 and 3:1 *o*AP:Fe(III) molar ratios. Slower rates of particle growth/agglomeration were observed for the 1:2 and 1:3 molar ratios. Fig. 1j shows that the maximum rate of particle growth/agglomeration was observed at 1:1 *o*AP:Fe molar ratio, which decreased with either reactant present in excess. This observation can be explained in light of the mechanism shown in Supplementary Scheme 2 and the mass yield results described above. It is likely that the pH affects the stability of the intermediates, and hence, the overall reaction rate for *o*AP:Fe molar ratio greater than 1. For molar ratios less than 1, the initial concentration of the reactant *o*AP is rate-limiting.

Higher ionic strength adjusted using 1 M ammonium sulfate, (NH_4_)_2_SO_4_, as the background solution appeared to increase the rate of particle growth/agglomeration for 1:3 and the 1:2 *o*AP:Fe molar ratio (i.e., excess iron region) by a factor of 4 relative to rates observed at low ionic strength using 0.01 M KCl (see data in Supplementary Figure 5 versus Fig. 1g). The salt content of aerosol liquid water in sea spray aerosols (or upon evaporation of water in cloud droplets) was found to affect the kinetics of certain reactions^72^ and increase or decrease the partitioning/solubility of nonelectrolytes, particularly organics, in processes referred to as ‘salting in’ or ‘salting out’^73,74^. For example, for organics in (NH_4_)_2_SO_4_ and sodium chloride solutions, Wang et al.^75^ reported that for the same neutral organic compound, (NH_4_)_2_SO_4_ has a higher ‘salting out’ effect than NaCl. As emphasized by the authors^75^, (NH_4_)_2_SO_4_ does not only affect the solvation of organic compounds in water, but could influence the reactive fate of some solvated organics. Since both Fe(III) and *o*AP are present at 1000x lower concentration than ammonium sulfate, it is likely that in our studies here, the ‘salting out’ effect of *o*AP in the 1:3 and 1:2 ratio by ammonium sulfate resulted in concentrating the organics with Fe(III) in a solvation cage leading to enhancements in the rate of particle growth/agglomeration. This process might be facilitated by the presence of four and five water molecules complexed to Fe(III) since the Fe(SO_4_)^+^ and Fe(SO_4_)_2_^−^ are the dominant aqueous phase species^56^. This finding warrants further investigation. The next section describes in detail the optical properties of the soluble and insoluble products from the reactions of aminophenols with Fe(III).

### Optical properties of soluble and insoluble products

**Fig. 2:**
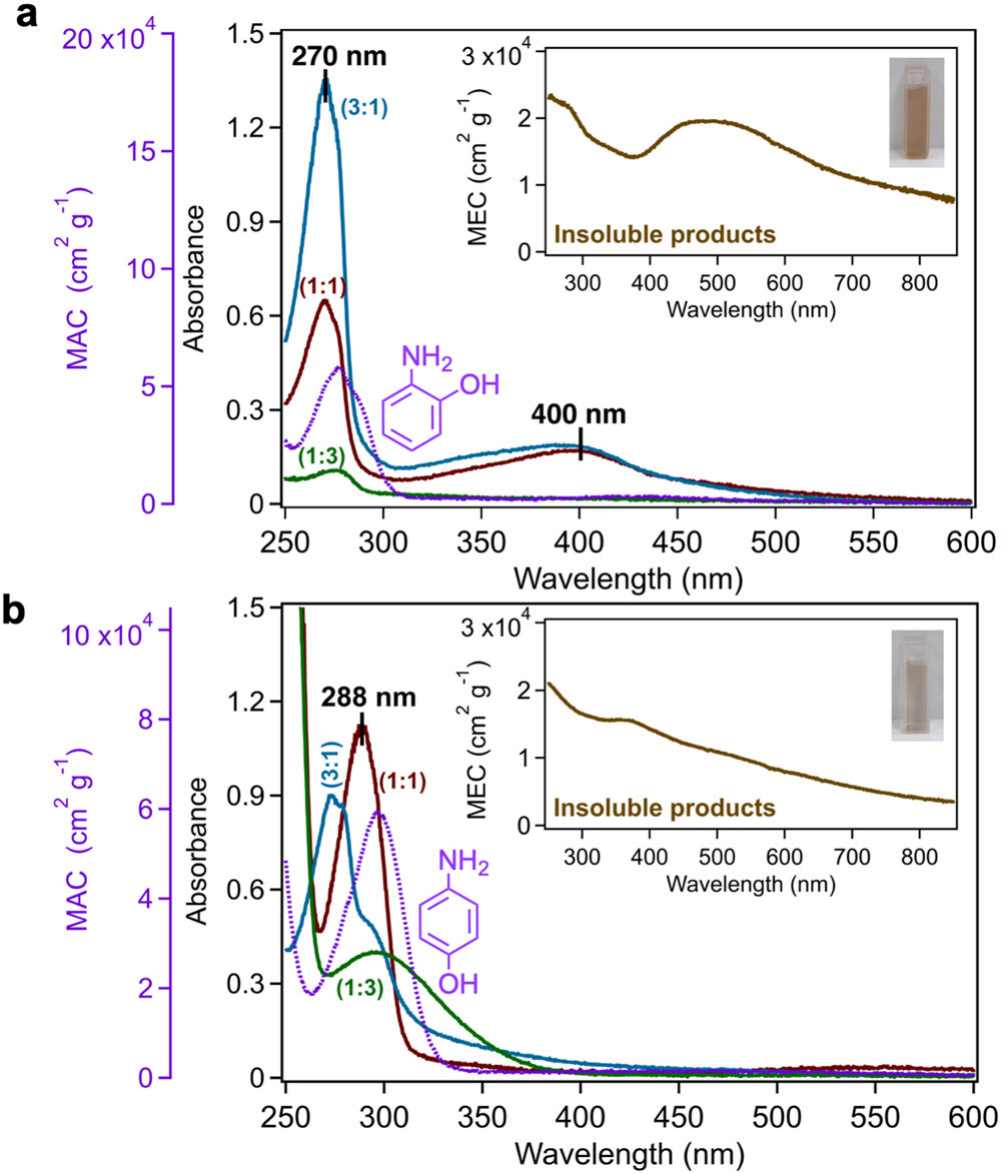
Electronic properties of soluble and insoluble reaction products. UV–vis absorbance spectra for the filtrates following overnight reaction of (**a**) *ortho*-aminophenol (*o*AP) and (**b**) *para*-aminophenol (*p*AP) with Fe(III) using the same concentrations listed in Fig. 1 for different aminophenol:Fe molar ratios, (pH 3–4.5). The MAC spectra for the aminophenol reactants are also shown as references. The insets are the MEC spectra for diluted aqueous slurries of the insoluble solid products using particle concentrations (**a**) 4.5 × 10^−5^ and (**b**) 8 × 10^−5^ g cm^−3^, respectively.

Fig. 2 shows the UV–visible absorbance spectra for the soluble products in the filtrates from the reaction of (a) *o*AP and (b) *p*AP with Fe(III) in the pH range 3–4.5. For reference, the absorbance spectra of standard *o*AP (1.6 × 10^−4^ M) and *p*AP (3 × 10^−4^ M) solutions are also shown, which were converted to mass-normalized absorption coefficient (MAC)^7^ spectra per Eq. (1):

1 $${{{{{{\rm{MAC}}}}}}}\left(\lambda \right)({{{{{{{\rm{cm}}}}}}}}^{2}{{{{{{\rm{g}}}}}}}^{-1})=\frac{A\left(\lambda \right)\cdot {{{{{{\rm{ln}}}}}}}(10)}{l\left({{{{{{\rm{cm}}}}}}}\right)\cdot {C}_{{{{{{{\rm{mass}}}}}}}}({{{{{\rm{g}}}}}}\,{{{{{{{\rm{cm}}}}}}}}^{-3})}$$

where *l* is the sample path length and *C*_mass_ is the mass concentration of the aminophenol. The spectra show π→π* transitions of the aromatic ring at 277 and 296 nm for *o*AP and *p*AP. The spectra for the filtrates are different than the standard solutions and from each other suggesting that soluble products have distinct structures and amounts depending on the aminophenol:Fe ratio. Chromatographic and mass spectrometric analyses of these products are presented in the next section. Here we emphasize that some of these products have absorbances in the near UV and visible (λ > 290 nm) wavelengths that overlap with the actinic flux, most notably the broad feature at 400 nm from the reactions of *o*AP with Fe(III) at the 3:1 and 1:1 *o*AP:Fe molar ratios. The spectrum of the filtrate from the reaction containing 2:1 *o*AP:Fe molar ratio is identical to those observed for the aforementioned ratios, and hence not shown here. This broad feature is absent from the 1:3 *o*AP:Fe molar ratio suggesting undetectable amounts of the soluble reaction products. This broad feature at 400 nm together with nitrogen from *o*AP suggests the presence of a quinone imine as we previously analyzed^56^. The MAC values in the 300–500 nm range between (1–2) × 10^4^ cm^2^ g^−1^ are comparable to those from primary BBOA (10^3^–10^4^ cm^2^ g^−1^)^76^. The next section describes in detail the analysis of soluble products in the filtrate solutions using UHPLC-UV-MS and ion chromatography MS.

Moreover, the insets in Fig. 2 show mass-normalized extinction coefficient (MEC) for the colored solid products calculated using Eq. (2):

2 $${{{{{{\rm{MEC}}}}}}}\left(\lambda \right)({{{{{{{\rm{cm}}}}}}}}^{2}{{{{{{\rm{g}}}}}}}^{-1})=\frac{{{{{{{\rm{ln}}}}}}}\left(10\right)\cdot {{{{{{\rm{measured}}}}}}}\,{{{{{{\rm{decadic}}}}}}}\,{{{{{{\rm{extinction}}}}}}}(\lambda )}{l\left({{{{{{\rm{cm}}}}}}}\right)\cdot {{{{{{\rm{particle}}}}}}}\,{{{{{{\rm{mass}}}}}}}\,{{{{{{\rm{concentration}}}}}}}({{{{{\rm{g}}}}}}\,{{{{{{{\rm{cm}}}}}}}}^{-3})}$$

The spectra show that scattering from the suspended particles is dominant with wavelength-dependent features. The broad band between 300 and 500 nm in the insets of Fig. 2 is in line with the analysis of the nitrogen-containing functional groups described above from the ATR-FTIR spectra of the solid films. The hygroscopic properties of these colored solid insoluble products are presented in detail below.

### Identification of soluble reaction products

**Fig. 3:**
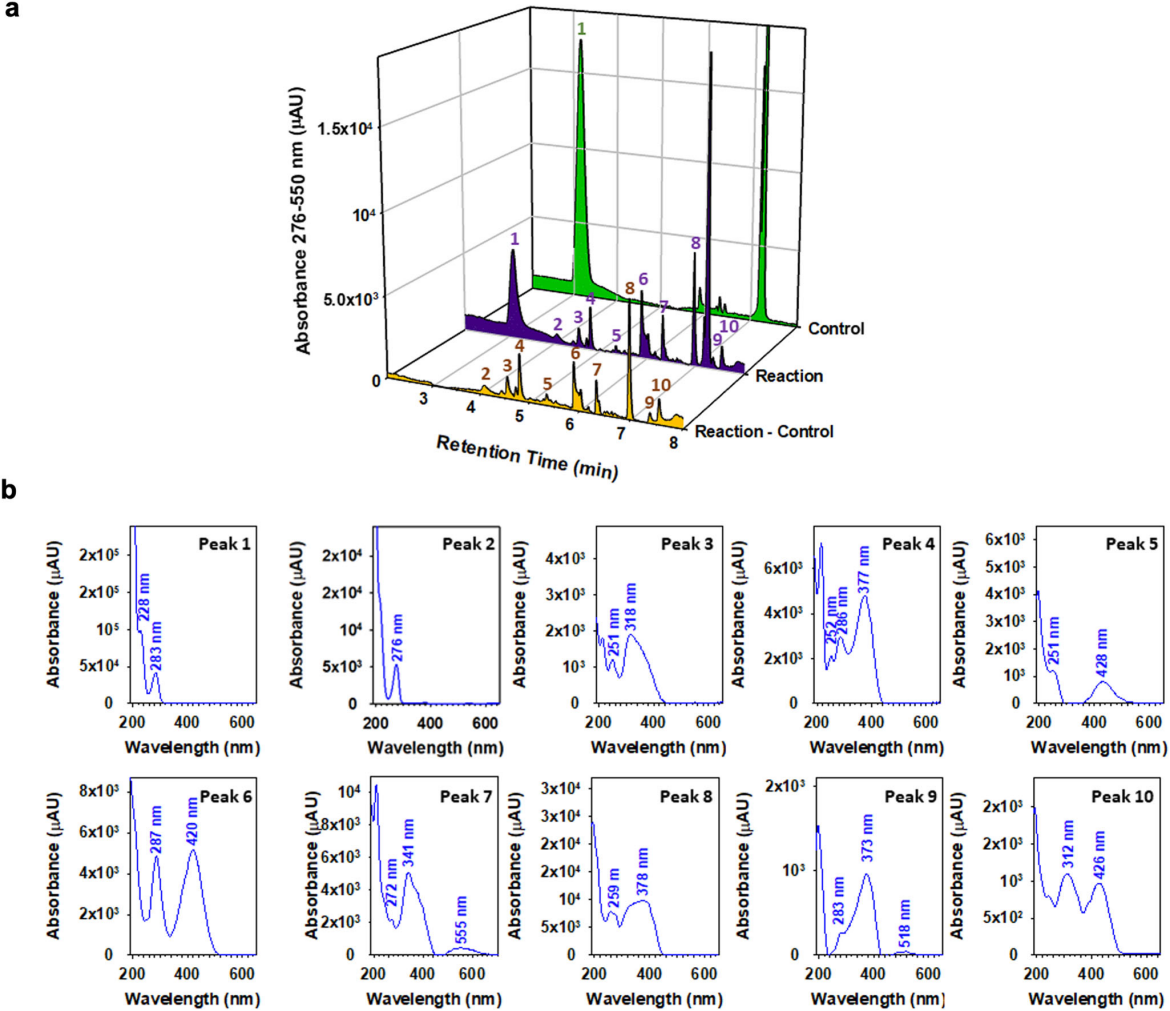
Chromatographic analysis of soluble products from the reaction of *ortho*-aminophenol (*o*AP) with Fe(III). **a** UV–visible (276–550 nm) chromatogram with additional ESI/MS(+) detection for the filtrates following the overnight reaction of (purple) *o*AP (labeled as peak 1) with Fe(III) in a 3:1 ratio, (green) a control in the absence of Fe(III), and (yellow) the remaining positive signal after subtraction of the control to the reaction. Labeled product peaks 2–10 are discussed in the text. Detailed reaction conditions and concentrations are the same listed in Fig. 1. **b** Extracted UV–visible spectra of peaks 1 through 10 in the chromatograms of Fig. 3.

Figure 3 displays the UV–visible chromatogram for the wavelength range 276–550 nm with additional ESI/MS(+) detection for the reaction of *o*AP (purple filled trace) with Fe(III) in a 3:1 ratio. The wavelength range was selected to illustrate the absorption of relevant products from the near UV to the center of the visible spectrum. Additionally, the use of an ESI/MS(+) detector allow us to extract the *m/z*^*+*^ values for the corresponding chromatographic peaks reported. Further, analysis of the previous sample was performed after spike addition with 4-nitrophenol, catechol, hydroquinone, and 4,4’-dihydroxybiphenyl. The green filled trace in Fig. 3 corresponds to a control without Fe(III), while the subtracted chromatogram for the reaction minus its control is presented with yellow color to help guide the eyes through a total of ten peaks labeled in Fig. 3. The retention time (*t*_r_) of *o*AP reactant labeled as peak 1 in Fig. 3a is *t*_r_ = 3.08 min. The absorption maxima of *o*AP occur at maximum wavelengths of λ_max_ = 201, 230 and 284 nm (Fig. 3b). The positive mode ionization of *o*AP shows adducts of the parent molecule (M) with H^+^ (M + H^+^) at *m/z*^*+*^ 110.14 (M = C_6_H_7_ON), and also with acetonitrile (M + CH_3_CN + H^+^) at *m/z*^*+*^ 151.10. For product identification, multiple formulas could be assigned to the many isomers that exist for a given mass. The nitrogen rule was taken into consideration when assigning even and odd molecular formulas. No additional constrains were used to limit the number of atoms or double bond equivalents. More details are provided in the Supplementary Methods section.

Peak 2 in Fig. 3a corresponds to catechol, an important product eluting at *t*_r_ = 4.07 min, which forms an ion at *m*/*z*^+^ 227.15. This mass-to-charge ratio corresponds to an adduct of M + 2 NH_4_^+^ + 2 CH_3_CN–H^+^. The elemental formula of the cationic peak adduct is C_10_H_19_O_2_N_5_^+^, as confirmed for the spike addition of catechol to the same sample that increased the peak height without changing peak shape and position. The extracted UV–visible spectrum for peak 2 is displayed in Fig. 3b with a maximum at 276 nm, as expected for catechol. Reaction R1 in Supplementary Scheme 3a indicates the attack of OH radicals, generated in the presence of Fe(III) (among other reactions), to substitute the –NH_2_ group of *o*AP and generate catechol^43^ Alternatively the substitution of an H atom by HO should (represented in reaction R2, Supplementary Scheme 3a) should create 3-aminocatechol (or 4-aminocatechol). Peak 3 in Fig. 3a elutes at *t*_r_ = 4.54 min with *m*/*z*^+^ 335.17 and shows absorptions in Fig. 3b at 318 nm (with a shoulder to the right extending to ~430 nm) and 251 nm. Such ion C_16_H_23_O_4_N_4_^+^ can result from the coupling of *o*AP and catechol (e.g., 3′-amino-[1,1′-biphenyl]-3,4,4′-triol, Reaction R3 in Supplementary Scheme 3a), which forms an adduct with two acetonitrile molecules, a water and an ammonium ion.

Peak 4 in Fig. 3a with *t*_r_ = 4.79 min absorbs at 252, 286 and 377 nm with a tail that extends to 400 nm in Fig. 3b and forms an ion at *m*/*z*^+^ 234.23. One general formula to describe such an ion is C_12_H_16_O_2_N_3_^+^, which results from an ammonium adduct to a dimer of *o*AP (e.g., 3,3’-diamino-[1,1’-biphenyl]-4,4’-diol, Reaction R4 in Supplementary Scheme 3a). Peak 5 elutes at *t*_r_ = 5.34 min with absorption bands centered at 215 and 428 nm for an ion C_12_H_13_O_3_N_2_^+^ at *m*/*z*^+^ 233.21 for a proton adduct of a coupling dimer between *o*AP with 3- or 4-aminocatechol (a product from OH attack to *o*AP such as 2,3’-diamino-[1,1’-biphenyl]-3,4,4’-triol, Reaction R5 in Supplementary Scheme 3a). Peak 6 elutes at *t*_r_ = 5.89 min and absorbs strongly with λ_max_ = 420 nm forming an ion *m*/*z*^+^ 340.11 for a proton adduct of a trimer with two *o*AP and one 3- or 4-aminocatechol molecules (e.g., 3,3’,3”-triamino-[1,1’:2’,1”-terphenyl]-4,4’,4”,5’-tetraol, Reaction R6 in Supplementary Scheme 3a). Instead, peak 7 (*t*_r_ = 6.32 min) displays an extended weak absorption at λ_max_ = 555 nm for an ion at *m*/*z*^+^ 336.27, which is 4 Da lighter than the previous trimer suggesting the formation of two new rings among two *o*AP and one 4-aminocatechol molecules. Such a structure for Peak 7 can be represented by the molecule of 2-amino-8-hydroxy-14,14a-dihydrobenzo[5,6][1,4]oxazino[3,2-b]phenoxazin-3(12H)-one (Reaction R7 in Supplementary Scheme 3a).

Peak 8 elutes at *t*_r_ = 7.23 min in Fig. 3a for a proton adduct ion C_18_H_13_O_4_N_2_^+^ at *m*/*z*^+^ 321.19 showing a broad band with λ_max_ = 378 nm (extending to ~445 nm) in Fig. 3b. Peak 8 can be described by the structure of 12,14-dihydrobenzo[5,6][1,4]oxazino[3,2-b]phenoxazine-2,3-diol (Reaction R8 in Supplementary Scheme 3a) originating from the coupling of two *o*AP and one catechol molecules. Peak 9 is shown at *t*_r_ = 7.36 min (Fig. 3a) for a proton adduct ion C_18_H_11_O_6_N_2_^+^ at *m*/*z*^+^ 351.20 that results in a strong and broad band with λ_max_ = 373 nm and another one at 518 nm. Peak 9 could be described by the structure 2,6,8-trihydroxybenzo[5,6][1,4]oxazino[3,2-b]phenoxazin-3(5aH)-one (Reaction R9, Supplementary Scheme 3a), a cyclic trimer among two 3-aminocatechol and one catechol molecules. Peak 10 (*t*_r_ = 7.54 min) with λ_max_ = 312 and 426 nm and *m*/*z*^+^ 319.17 for the ion C_18_H_11_O_4_N_2_^+^ is a proton adduct of similar origin to the previous peaks as described by the molecule of 2-hydroxybenzo[5,6][1,4]oxazino[3,2-b]phenoxazin-3(5aH)-one (Reaction R10, Supplementary Scheme 3a) produced from two *o*AP and one catechol molecules.

Supplementary Figure 6a shows the UV–visible chromatogram between 276 and 550 nm for the reaction of *p*AP (purple) with Fe(III) followed by the interpretation of these peaks per the suggested mechanism in Supplementary Scheme 3b. In summary, the analysis of the soluble reaction products revealed Fe-catalyzed formation of ring coupling products of *o*- and *p*-aminophenols and their primary oxidation products. Given their structure and optical properties, these products would be reactive under day- and night-time conditions in the bulk and at interfaces, which is worth exploring.

### Efficiency of cloud condensation nucleation

**Fig. 4:**
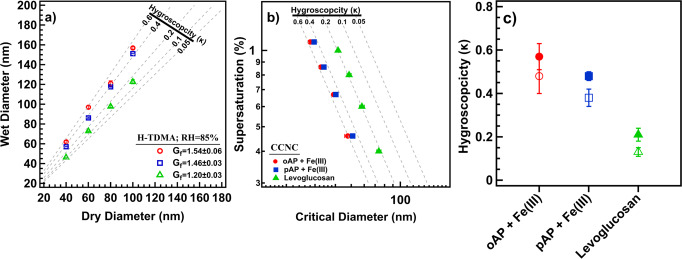
The cloud condensation nuclei activity of oligomers from the *ortho*-aminophenol (*o*AP)+Fe(III) reaction (red circles), *para*-aminophenol (*p*AP)+Fe(III) reaction (blue squares), and levoglucosan (green triangles). **a** The wet diameter growth versus initial dry diameter of particles exposed to a subsaturated environment (85% RH) from H-TDMA measurement. The average *G*_*f*_ for each material is also reported. Gray dashed lines show theoretical *κ*-Köhler values. **b** The average critical diameter versus supersaturation from CCNC measurement (closed symbols). Gray dashed lines show theoretical *κ*-Köhler values. A decrease in critical diameter size at a constant supersaturation indicates an increase in CCN and droplet activity. **c** Summary of Köhler theory hygroscopicity parameter, *κ*, acquired from CCNC (closed symbols) and H-TDMA (open symbols) measurements. Each point represents an average of 10 points. Error bars show standard deviation.

The water uptake and droplet formation by insoluble oligomers from the reaction of *o*AP and *p*AP with Fe(III) were investigated under subsaturated and supersaturated conditions using a Humidified Tandem Differential Mobility Analyzer (H-TDMA) and a Cloud Condensation Nuclei Counter (CCNC), respectively. Fig. 4 summarizes the water-uptake of these polymers in conjunction with levoglucosan—a prominent proxy for BBOA. In both sub- and supersaturated conditions, oligomers from the *o*AP + Fe(III) reaction were slightly more hygroscopic than from the *p*AP + Fe(III) reaction, and both types were more hygroscopic than levoglucosan. Organic particles exposed to 85% RH swelled and the measured growth factor, *G*_*f*_ (derived from the ratio of the wet to initial dry particle diameter), was 1.54 ± 0.06, 1.46 ± 0.03, and 1.20 ± 0.03 for particles from *o*AP + Fe(III), *p*AP + Fe(III), and levoglucosan, respectively (Fig. 4a). A study by Chan et al.^77^ investigated the water uptake of a series of amino acids and organics derived from biomass burning and reported the growth factors, at 85% RH, to range from 1.19–1.33. This growth factor range agrees with our biomass burning derived levoglucosan measurements, but not with *o*AP + Fe(III), *p*AP + Fe(III). The high growth factors measured for *o*AP + Fe(III), *p*AP + Fe(III) can be attributed to the presence of high-density hydrophilic functional groups in their polymeric structures. Previous literature has ascribed the high hygroscopicity measured for insoluble polymers due to the presence of hydroxyl groups in their structures^78^. In addition, The high surface area of their polymeric structures is conductive for water condensation which can drive droplet growth as reported by Gohil et al.^79^. At supersaturated conditions, oligomers from the *o*AP + Fe(III) and *p*AP + Fe(III) continued to grow, and the dry particle critical diameters (*D*_*d*_) were measured at four constant supersaturations (0.4–1.1%). At each supersaturation, the critical diameters increased from the *o*AP + Fe(III) to *p*AP + Fe(III) to levoglucosan (Fig. 4b). A smaller *D*_*d*_ at a constant supersaturation signifies an enhancement in CCN activity.

The average single hygroscopicity parameter *κ*-value derived from Köhler theory at subsaturated $({\kappa }_{{G}_{f}})$ and supersaturated conditions (*κ*_CCN_) is also reported (Fig. 4c). Under both subsaturated (H-TDMA) and supersaturated conditions (CCNC), oligomers from the *o*AP + Fe(III) were slightly more hygroscopic than those from the *p*AP + Fe(III) with ${\kappa }_{{G}_{f}}$ values of 0.48 ± 0.08 and 0.38 ± 0.04, respectively. The ${\kappa }_{{G}_{f}}$ value for Levoglucosan is 0.13 ± 0.02 and consistent with previously reported values^80,81^. For comparison, *κ*_CCN_ values for the oligomers from the *o*AP + Fe(III), *p*AP + Fe(III), and levoglucosan were 0.57 ± 0.06, 0.48 ± 0.02, and 0.21 ± 0.03, respectively.

For both oligomer types from the reaction of *o*AP + Fe(III) and *p*AP + Fe(III), the *κ*_CCN_ values are greater than their ${\kappa }_{{G}_{f}}$ values (Fig. 4c). Previous studies have attributed differences of ${\kappa }_{{G}_{f}}$ and *κ*_CCN_ values to surface tension depression of a pure water droplet^81–83^ and adsorption-driven droplet growth^78,84^. Surface tension pendant droplet measurements were conducted for both oligomer types in range of 0.3–2.0 g L^−1^ (Supplementary Figure 7) and both materials exhibited no surface activity within the measured range. Thus, the differences in *к* values are likely attributed to adsorption-dominated water uptake of insoluble and partially soluble aerosol droplet growth^78,84^. Overall, the measured values of the insoluble oligomer materials from aminophenols are consistent with more complex BBOA hygroscopicity values. The water uptake of BBOA is known to vary between (0 to 0.6) and the *κ* values reported here for oligomers under sub- and supersaturated conditions are within range of previously published literature values of direct biomass emissions^5,85–88^. Here, the measured oligomer *κ*-values are greater than 0.3 and less than 0.6. In addition, when comparing the CCN efficiencies of these oligomers relative to levoglucosan, we observe that these nitrogen-containing oligomers are more hygroscopic than levoglucosan. Köhler theory predicts water uptake to be inversely correlated with molecular weight and this relationship has been previously illustrated in literature with various organic aerosols^81,89^. Since the insoluble oligomers from the reaction of *o*AP + Fe(III) and *p*AP + Fe(III) are shown to have a higher molecular weight than levoglucosan (see above), other factors need to be considered in explaining their hygroscopic properties. Recent work^78^ showed that organic polymeric compounds found in BBOA may be more hygroscopic than previously thought. A study by Malek et al.^78^ investigated the hygroscopicity of two relevant and biomass burning based insoluble polymeric aerosols: polycatechol and polyguaiacol and found their *к-*values to range from 0.03 to 0.25. Their hygroscopicity was attributed to their chemical structure, specifically the presence of hydroxyl groups. Here, the *к*-values of the oligomers from the reaction of *o*AP + Fe(III) and *p*AP + Fe(III) are above 0.3. This can be attributed to the presence of a high density of hydriphilic carbonyl groups within their polymeric structure. Additionally, the high water uptake can be also explained by the polymers’ propensity for organic aerosol swelling analogous to water uptake of hydrophilic polymer substances^90,91^. Furthermore, previous literature^92,93^ also showed organic compounds containing sulfates or nitrates have enhanced hygroscopicity compared to organic carbohydrates. This enhancement in hygroscopicity is comparable to what we observe with *o*AP + Fe(III) and *p*AP + Fe(III). Peng et al.^94^ investigated the hygroscopicity of organosulfate species (sodium methyl sulfate, sodium ethyl sulfate, and sodium octyl sulfate) and found κ values to range between 0.459–0.206. In addition, Estillore et al.^95^ examined the water uptake of more than 10 organosulfates using H-TDMA, and reported the growth factor range to be between 1.30–1.50 The results of these two reports are comparable to the water uptake results obtained in this study. Hence, nitrogen-containing oligomers formed here offer a fascinating insight on the structural composition and their effect on water uptake. Thus, our results indicate that nitrogen-containing organic oligomers in BBOA may be as efficient as inorganic and/or organosulfate aerosol-cloud seeds from biomass sources.

### Dust-catalyzed formation of aminophenol oligomers a function pH

**Fig. 5:**
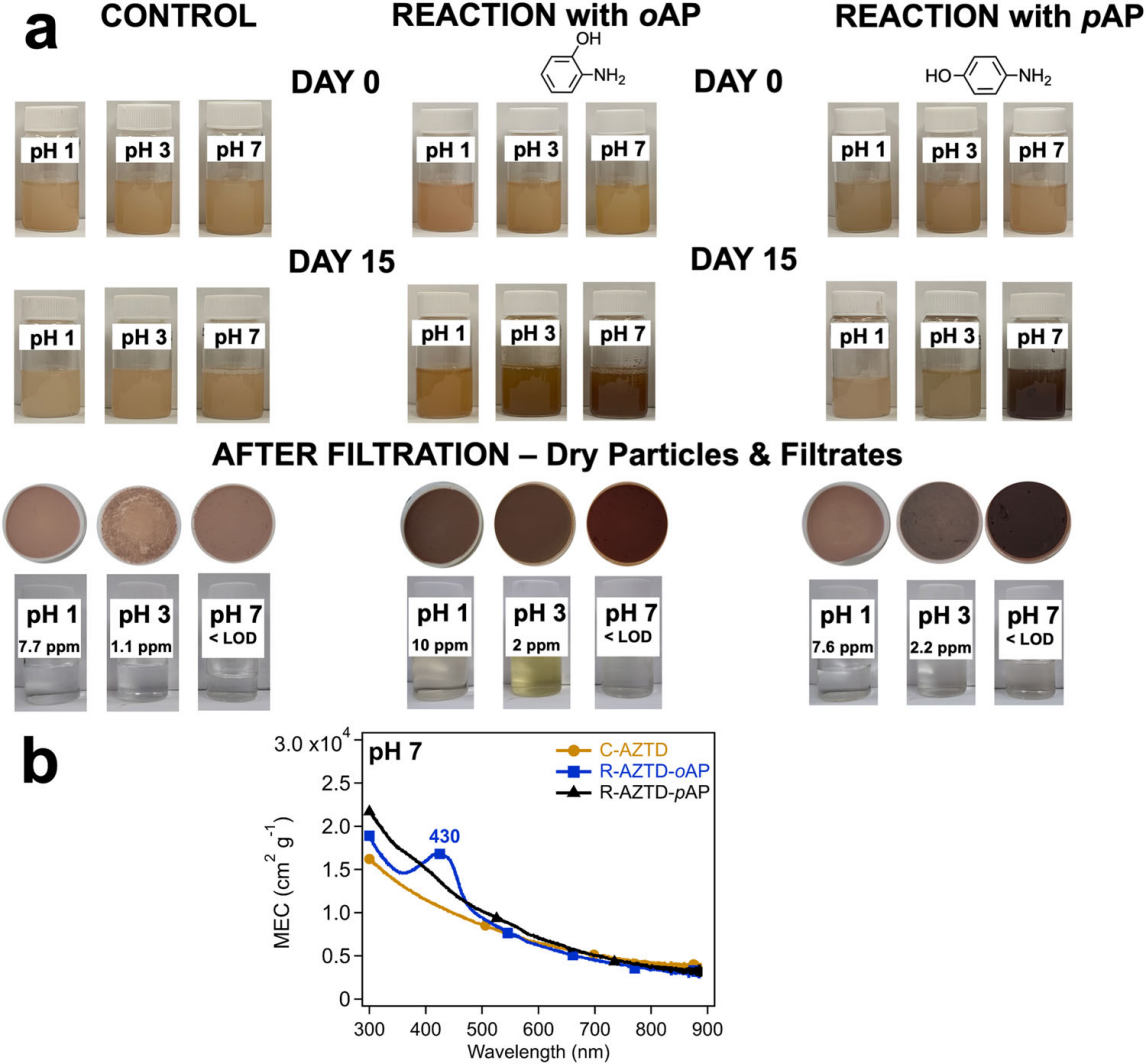
Reactivity of aminophenols with AZTD under simulated dust aging. **a** Digital photographs of control (left), reaction with *ortho*-aminophenol (*o*AP, middle), and *para*-aminophenol (*p*AP, right) vials containing AZTD as a function of pH and time (0 and 15 d) of simulated atmospheric aging time. Similar photos for days 2 and 7 are shown in Supplementary Figure 8. These samples were used for electron microscopy and elemental mapping. The pH values shown in the label of each vial refers to the starting pH of the slurry. At the end of day 15, the slurries were filtered, and the corresponding filters and filtrates are shown below each vial. The concentration of DFe in the filtrates after 15 d in the filtrates of are listed as well. The limit of detection was determined to be 0.06 ppm (mg L^−1^). **b** MEC spectra as a function of wavelength for diluted AZTD slurries at pH 7 for the control (no organics added) and reacted particles with *o*AP and *p*AP for 15 d. The absorbance and MAC spectra of filtrates at pH 3 are shown in Supplementary Figure 8.

To further show the atmospheric relevance of our work, simulated dark dust aging experiments were carried out using AZTD as the source of iron with and without dissolved *o*AP and *p*AP as a function of pH over two weeks. AZTD has abundant Lewis acid sites^96,97^ that act as electron acceptors including Fe(III). Figure 5 and Supplementary Figure 8 show photographs of control and reaction vials with time. The reaction conditions at pH 1 and 3 simulate acid-promoted dissolution in atmospheric aerosol particles that increase the concentration of dissolved iron (DFe)^98^. At pH 1, the average (±1σ < 8%) of triplicate measurements of DFe in all filtrate vials equals 7.7, 10, and 7.6 ppm for the control, reaction with *o*AP, and *p*AP, respectively. This result suggests that *o*AP slightly enhanced the dissolution of iron by 1.3x than *p*AP. At pH 1, below the first pK_a1_ for *o*AP, the protonated -NH_3_^+^ substituent is present with the -OH group on the benzene ring. Also, most surface sites on the minerals in AZTD are positively charged due to points of zero charge below 7 (Table S2 in the SI of ref. ^99^). *o*AP forms a complex with iron(III)(hydr)oxides in AZTD in a monodentate fashion via the -OH group with -NH_3_^+^ stabilized by hydrogen bonding with neighboring sites (Supplementary Scheme 4)^100^. This type of surface complexation was also observed for catechol on hematite under acidic conditions^101^. The formation of this monodentate complex enhances electron transfer to iron surface sites that results in releasing Fe(II) to solution and the formation of a phenoxide radical. *p*AP was reported to adsorb poorly on hematite^100^ due to the location of the -NH_3_^+^ substituent in the *para* position (Supplementary Scheme 4), which explains the similarity of DFe to the control confirming the dominant role of the proton-driven dissolution mechanism over ligand-promoted dissolution.

Increasing the pH to 3 decreased the amount of DFe in all vials. However, the reaction filtrates containing *o*AP and *p*AP had nearly 2x DFe relative to the control filtrate. This result suggests the decreased efficiency of proton-promoted dissolution relative to pH 1. It also suggests that increasing the pH to values closer to the pK_a_ of *o*AP and *p*AP (Supplementary Scheme 1) is enhancing iron dissolution via ligand-promoted mechanisms. At pH 3, surface sites on sanidine, albite and quartz in AZTD are negatively charged since their point of zero charge is below 3^102,103^. It is likely that -over the time scale of our experiment- these negatively charged surface sites enhance the deprotonation of the -NH_3_^+^ substituent on *o*AP leading to enhanced adsorption and electron transfer to iron-containing minerals in AZTD that result in releasing Fe(II) to the solution (Supplementary Scheme 4). The slurry containing *p*AP at pH 3 appears slightly darker compared to pH 1, and not as dark as that containing *o*AP at pH 3. This observation clearly shows the lower reactivity of *p*AP compared with *o*AP in oxidative coupling at pH 1 and 3, which is consistent with its adsorption capacity and oxidative oligomerization reactivity on hematite^100^.

At pH 7, DFe values are below the limit of detection of the instrument due to the significant decrease in acid-promoted dissolution kinetics. Reaction slurries containing the aminophenols appear much darker than the control slurry. Both aminophenols are neutral and have their maximum adsorption capacity at pH 7^100^. The oxidative oligomerization of *o*AP and *p*AP at pH 7 is catalyzed by surface Fe leading to the darkening of AZTD with time. Fig. 5b shows the MEC spectra for the control and reacted slurries. These spectra show the dominance of the scattering components with absorbance features around 430 nm observed for the reacted slurries compared to the control assigned to the quinone imine moieties. These features are similar to those contributing to the absorbance feature shown in the inset of Fig. 2a for the MEC spectrum of the insoluble product from the aqueous phase oligomerization of *o*AP with dissolved Fe(III).

Upon filtration following the 15 d of simulated dust aging, photographs of the dry filters in Fig. 5a show that AZTD particles from reaction vials are darker than the control, except pH 1 for *p*AP reaction. Since the filtrates are transparent, the dark brownish to black color observed in the AZTD slurries was due to the formation of insoluble products associated with reacted AZTD particles. Supplementary Figure 9 shows STEM images of control and reacted particles from the slurries at pH 7 coupled with % carbon, % oxygen, and % iron. These images show that the carbon content of the reacted AZTD particles is higher by a factor 4–10 than the control particles, oxygen content is slightly smaller in the reacted particles compared to the control, and that iron distribution is not uniform. Particle morphologies in the STEM images suggest that oligomerization products are coating the AZTD particles or agglomerated near them. Since oxygen content in the organic coating is relatively smaller than in the mineral content of AZTD particles, the slight reduction in % oxygen supports the above interpretation.

In addition, Supplementary Figure 8b shows the absorbance (left axis) and MAC spectra (right axis) for the filtrates at pH 3 of the control (C-AZTD) and reacted samples (R-AZTD-*o*AP and R-AZTD-*p*AP). The MAC spectra were calculated using the initial mass concentration of *o*AP and *p*AP of 1 mM (1.1 × 10^−4^ g mL^−1^). The MAC spectrum of R-AZTD-*o*AP filtrate shows similar features to the one shown in Fig. 2a for the soluble products from the aqueous phase reactions between *o*AP and Fe(III) at the (1:1) and (3:1) *o*AP:Fe molar ratios. Similarly, the MAC spectrum of R-AZTD-*p*AP filtrate in Supplementary Figure 8b is similar to the one shown in Fig. 2b for the soluble products from the aqueous phase reactions between *p*AP and Fe(III) at the (3:1) *p*AP:Fe molar ratio. These results suggest that our simulated AZTD aging experiments reflect excess organics relative to dissolved iron reaction conditions. Importantly, the proposed reactions of soluble *o*AP and *p*AP with Fe(III) result in absorption features of oligomers formed between the reactants and their primary oxidation products that explain the registered changes of MAC and MEC (particularly of peaks 5, 6, and 10 in Fig. 3b) reported above.

## Conclusions

Aminophenols are redox active organic molecules that contribute to the amine content in atmospheric aerosol particles from different sources. These compounds are water-soluble and have been shown to undergo oxidative coupling that leads to forming soluble and insoluble oligomers and polymers. The current picture of mineral dust aging is that iron dissolution is efficient under acidic conditions due to the uptake of acidic gases and complexation with oxalate. Our results here show that under aerosol and cloud reaction conditions of pH 1–7 and ionic strength from 0.01 to 1 M, simulated night-time chemistry of dissolved iron catalyzes oxidative oligomerization of aminophenols leading to the formation of soluble and dark brown to black insoluble products containing reduced nitrogen. The insoluble nitrogen-containing oligomers are as efficient in water uptake and cloud condensation as inorganic and/or organosulfate aerosol from biomass sources. The heterogenous iron-catalyzed chemistry shown here changes the morphology and optical properties of dust over atmospherically relevant timescales that simulate long range transport. Our studies here were  conducted using single organic compounds instead of a mixture of organic components to gain insights into reaction mechanisms. The single-component systems offer valuable knowledge with respect to how chemical structure impact reactivity with Fe(III). The dissolved Fe(III) and organic concentrations are atmospherically relevant for air masses containing aged dust from long range transport over urban areas and aim to simulate reactions in deliquesced aerosol liquid water. In real aerosol systems, aminophenol concentrations are source-dependent and might be lower than the ones used here, especially in remote areas. Also, there are other organic and inorganic components that can complex Fe(III) and affect its reactivity. For the experiments using 1 M ammonium sulfate, which is about 1000x higher concentration than Fe(III) and the aminophenols, oligomerization reactions took place despite sulfate being a strong chelating agent for Fe(III). This result is similar to what we observed earlier for catechol^56^ suggesting the high efficiency of redox chemistry in multicomponent systems. Ongoing studies in our labs investigate the role of ubiquitous C2-C6 dicarboxylic acids on the extent of these oligomerization reactions.

Our results provide insights into our understanding of mineral dust redox reactivity and its impact on climate forcing and hygroscopic properties. The key variables that affect the radiative forcing (heating or cooling effect) of dust include height of dust layer, particle size and aerosol optical depth^104^. Here, we show that dust aging chemistry due to reaction with redox active nitrogen-containing compounds may change the radiative forcing of dust aerosol to that of black carbon. It also shows that reaction products may enhance the hygroscopic properties of dust in cloud seeding due to changes to surface functional groups. Changes to dust morphology, mixing state, and chemical composition due to the chemistry we report here opens avenues for exploring changes to dust photochemical reactivity influenced by the nitrogen-containing oligomeric content. Given the wide variety of NOC in ambient aerosol particles and the analytical challenges of directly measuring their concentration and speciation, more field research is needed to confirm and evaluate the relevance and importance of these lab results in the atmosphere. In particular, collecting ambient particles in the fine and ultrafine size modes from urban/industrial geographical regions on the path of long-transported dust plumes would be most influenced by this type of chemical reactions.

## Methods

### Chemicals

All chemicals—listed in the SI—were used as received without further purification.

### Dynamic light scattering (DLS) experiments

DLS experiments were performed to monitor growth of insoluble polymeric materials forming in solution within the first hour of reaction time. Each reaction took place in a 20 mL vial containing 5 mL of a known concentration of the organic precursor prepared in either 0.01 M KCl or 1 M (NH_4_)_2_SO_4_ background solution. Additional details are listed in the SI.

### Insoluble product mass yield experiments

The mass yields of insoluble products were determined from 2 h reactions using 3:1 and 1:1 molar ratio organic reactant:Fe by weighing the filters before and after filtration followed by overnight drying followed by characterization. More details are in the SI.

### Spectroscopy and Chromatography analyses

Ultraviolet-visible (UV–vis) spectra of diluted control and reacted solutions and AZTD slurries were collected using a UV–vis spectrophotometer (Ocean Optics USB 4000) in a 1-cm quartz cuvette. Selected filtrate samples and the corresponding controls were analyzed after dilution in water (18.2 MΩ cm) with 5% acetonitrile (LC/MS grade) by 10 and 16.67 times, respectively, by UHPLC-UV-(ESI)MS (Accela 1250 with photodiode array and MSQ Plus detectors, Thermo Scientific) equipped with a C18 column (ZORBAX Eclipse Plus RR HD, 2.1 × 100 mm, 1.8 µm). More details are in the SI.

### CCN activity

Supplementary Figure 10 shows the experimental setup for the Hygroscopicity Tandem Differential Mobility Analyzer (H-TDMA)^105,106^ and Cloud Condensation Nuclei Counter (CCNC) measurements^107,108^ with more details in the SI.

### Acid-promoted dissolution experiments

These experiments were performed using AZTD and following a modified procedure from that reported by Link et al.^99^. More details are in the SI.

## Supplementary information

Supplementary Information

## Data Availability

The data that support the findings of this study are available from the corresponding authors upon reasonable request.
