# Supplementary material for: Reactivity of aminophenols in forming nitrogen-containing brown carbon from iron-catalyzed reactions
Source: Commun Chem. 2022 Sep 19;5:112. doi: 10.1038/s42004-022-00732-1 (PMC9814260; doi:10.1038/s42004-022-00732-1)
Supplement: Supplementary file 1 — Supplementary Information [file 42004_2022_732_MOESM1_ESM.pdf]

Supporting Information

Reactivity of aminophenols in forming nitrogen-containing brown carbon from iron-catalyzed reactions

Hind A. Al-Abadleh[1*], Fatemeh Motaghedi[1a], Wisam Mohammed[1a], Md Sohel Rana[2a], Kotiba A. Malek[3a], Dewansh Rastogi[3a], Akua A. Asa-Awuku[3*], and Marcelo I. Guzman[2*]

[1] Department of Chemistry and Biochemistry, Wilfrid Laurier University, Waterloo, Ontario N2L 3C5, Canada.
[2] Department of Chemistry, University of Kentucky, Kentucky 40506, United States.
[3] Department of Chemical and Biomolecular Engineering, University of Maryland, College Park, Maryland 20740, United States.

[*] Corresponding authors: H.A.A. (halabadleh@wlu.ca), A.A.A. (asaawuku@umd.edu), M.I.G. (marcelo.guzman@uky.edu). [a] These co-authors contributed equally to the experimental work.

Date: September 6, 2022

Supplementary Methods

Chemicals. *o*-aminophenol (99%, CAS: 95-55-6, Sigma-Aldrich), *p*-aminophenol (≥98%, CAS: 123-30-8, Sigma-Aldrich), iron(III) chloride hexahydrate (97%, CAS: 10025-77-1, Sigma-Aldrich), potassium chloride (KCl powder, 99.5%, EM Science), ammonium sulfate (ACS grade, CAS: 7783-20-2, EMD Chemicals), and hydrochloric acid (HCl 6 N, Ricca Chemical Company). Arizona test dust (Powder Technology, Inc., 0-3 μm nominal particle size, ~5% (wt/wt) Fe, composed of muscovite (42%), quartz (24%), albite (16%), kaolinite (2.8%), sanidine (7.3%), and calcite (6.2%) as determined using powder X-ray diffraction) was sonicated for 20 min in a 90:10% water:ethanol mixture followed by suction filtration and two cycles of washing with Milli-Q water (18.5 MΩ cm) only and subsequent filtration and drying in the oven prior to use. Aqueous phase solutions were prepared by dissolving the chemicals in Milli-Q water. All experiments with these chemicals were conducted under dark solution conditions by wrapping vials and beakers with aluminum foil in order to prevent competing photo processes. The pH was measured using a calibrated gel-filled or glass pH electrode (Orion).

Dynamic light scattering experiments. The timer started with the addition of 0.1 mL $FeCl_3$ of a known concentration. The final reaction pH and concentration of the organic precursor and $FeCl_3$ are listed in the text. Within the first minute of reaction, 3 mL were taken to the 1 cm disposable cuvette located in the DLS instrument, Malvern Zetasizer Nano (ZEN3600), to start the data collection. The settings in the data collection software were as following: dispersant (water), backscattering angle (-173° , default), viscosity (0.0082 cP), equilibrium temperature (25° C), the real refractive index of the material (1.604), imaginary refractive index (0.000), number of measurements (varied from 12-36), the number of runs for each measurements was set to 'automatic' so that parameters on the detector (e.g., measurement position, attenuator factor) were optimized for each experiment. A new disposable cuvette was used for each measurement, which was rinsed by ultrapure water prior to adding the solution mixtures. The standard deviation ($\pm 1\sigma$) in d was calculated to be around 30% from multiple experiments. Given the physics of light scattering in solutions containing particles, $\langle d \rangle$ recorded by the instrument as a function of reaction time reflected either particle growth or agglomeration. The pictures of vials were taken at the end time of the DLS experiments to show the color of the reaction solutions over shorter reaction times. These solutions were allowed to react for 24 h prior to filtration on nylon membrane filters (0.2 μm pore size, 25 mm diameter, EMD) to assess whether particle growth was too slow to be detected using DLS or no particles would form irrespective of time.

Insoluble product mass yield experiments. For these experiments, the reaction volumes were considerably scaled up (101 mL) in order to achieve easily measurable mass yields of particles, keeping the starting iron concentration the same in all experiments (1 mM). Using a standard suction filtration system, particles were collected on nylon membrane filters (0.2 μm pore size, 45 mm diameter, EMD). The collected particles were then washed with Milli-Q water several times prior to drying. The mass of the filters with the particles were measured to compare to the mass of the clean filters.

Insoluble particle preparation for characterization. The same procedure used for forming particles for mass yield experiments described above was used to obtain particles for characterization. The only difference is that no filtration took place. Instead, the slurries were

transferred to centrifuge tubes to separate the particles from the solvent. The first supernatant was discarded and Millipore water was added to resuspend the particles followed by another centrifugation step. This step was repeated three times. Then, concentrated slurries were left to dry in non-static weigh boats. Wood spatulas were used to collect the dry particles in microcentrifuge vials. These dry particles were then used for particle characterization using attenuated total reflectance Fourier transform infrared spectroscopy (ATR-FTIR), thermogravimetric analysis (TGA), and Matrix Assisted Laser Desorption Ionization Time of Flight Mass Spectrometry (MALDI-TOF-MS). For electron microscopy and hygroscopicity experiments, the particles resuspended in Millipore water were stored in the fridge till further use. These samples are mostly insoluble reaction products but the presence of residual soluble fraction cannot be excluded.

Attenuated total reflectance Fourier transform infrared spectroscopy (ATR-FTIR). Characterization of the insoluble oligomeric products using ATR-FTIR was completed by depositing a concentrated water/ethanol slurry of the particles onto a ZnSe crystal followed by drying overnight. Absorption spectra of these particles were obtained by referencing to the clean and dry ZnSe crystal.

Thermogravimetric analysis (TGA). TGA is a technique useful to provide insights into molecular structure relative to published reports." Dry particles were used for the TGA analysis, which was completed on a TGA Q500 from TA Instruments using air as the purge gas at the Analytical Lab Services of the at the University of Waterloo. The sample was heated from room temperature to 600°C at a rate of 20 °C min^{-1}.

Matrix Assisted Laser Desorption Ionization Time of Flight Mass Spectrometry (MALDI-TOF-MS). These experiments were conducted to obtain data on the structure of the insoluble colored oligomers using a Bruker Auto Flex Speed MALDI-TOF System equipped with a 2 kHz Nd:YAG UV laser (355 nm) at the Mass Spectrometry Facility of the University of Waterloo. A concentrated slurry of the oligomers were prepared in water only, with no matrix, from which samples were spot-deposited on the MALDI stainless steel sample plate forming thin films. The spots were dried in air at room temperature before being loaded to the MALDI-TOF-MS sample holder. Data were acquired in negative ion mode with laser power adjusted to result in the minimum visible damage to the sample. Scans of different spatial domains of the sample were accumulated to ensure reproducibility of mass features.

Chromatography analyses. An electrospray ionization (ESI) source served as the interface between the UHPLC and a calibrated mass spectrometer (MS). Chromatographically separated products were detected with a single quadrupole mass spectrometer accurate to 0.17 amu. The method has been previously described[1] and is briefly summarized next. Fifty microliters were injected with an autosampler into C_{18} column (ZORBAX Eclipse Plus RR HD, 2.1 mm× 100 mm, 1.8 μm) and analyzed by gradient elution during 16 min at a flow rate of 0.400 mL min^{-1} employing two pair of solvents, 1.0 mM ammonium formate in water (solvent A) or acetonitrile (solvent B) for ESI(+)/MS. The solvent gradient was kept isocratic for 2 min with 5% B, and then increased at a rate of 9.5% B per minute. The same samples were analyzed after spike addition with 4-nitrophenol (ACROS Organics, 100.0%), catechol (Sigma-Aldrich, 99.9%), hydroquinone (Sigma-Aldrich, 99.9%), and 4,4'-dihydroxybiphenyl (Alfa Aesar, 99.9%). %). Elemental formulas proposed in the work considered the possible molecular structures that can be generated

from the reactants (*o*AP and *p*AP), and the intermediate products identified with authentic standards, through their reactivity in known chemical pathways. The general formulas provided by Xcalibur were carefully analyzed based on known organic chemistry of the reactant and intermediates with the condition that the structures kept an acceptable ratio of hydrogen atoms to the number of carbon and oxygen centers. In other words, under the experimental oxidizing conditions of this work, the number of hydrogen atoms in a molecule that did not incorporate nitrogen cannot exceed that of the precursor molecule(s).

Electron microscopy experiments. (a) Oligomers. Transmission Electron Microscopy (TEM) was used to visualize nano-sized oligomers from the reaction of Fe(III) with *o*-aminophenol (*o*AP) and *p*-aminophenol (*p*AP). The oligomers were aerosolized first using a collision atomizer (TSI 3076) and then dried (RH < 5%) using diffusion dryers containing silica gel to prepare imaging samples. The dry particles were charged with a neutralizer (Kr-85, TSI 3077A), and then deposited onto an electrically grounded TEM grid (Cu, Lacey carbon coated, TED PELLA) for a duration of 4 h. The aerosol particles were then imaged using JEOL 2100 TEM (LaB_6 filament, University of Maryland) at an accelerating voltage of 200 kV and magnification range of 50-150 k. Exposure time of sample to electron beam was kept low (90 sec) to minimize sample damage caused by high energy electrons (1). Energy Dispersive X-Ray Spectroscopy (EDS) was also used to analyze the composition of aerosol particles, specifically for elements with higher atomic numbers such as Fe, Ca, Mg, Si, Al, and K. Carbon and copper elemental peaks are not reported as the TEM grids utilized were made of copper coated with lacey carbon. **(b) AZTD samples.** For imaging dust particles, High-Angle Annular Dark-Field Scanning Transmission Electron Microscopy (HAADF-STEM) (TEM) coupled with energy dispersive spectroscopy (EDS) analysis was used. The samples were spread on silicon nitride window grids from Norcada from water slurries. The experiments were performed in a Thermo Scientific Talos 200X operated at 200kV at the Canadian Centre for Electron Microscopy located at McMaster University. The images and the maps were acquired using the Velox program. The EDS maps were acquired in HAADF-STEM mode using the Velox program from Thermo Scientific. Over twenty images were collected for different particles deposited on multiple grids to ensure that images shown here are representative of the source slurries. For the quantification of the elements in the EDS maps, the software was carefully calibrated with standard samples.

CCN activity. *<u>(a) Subsaturated Measurements</u>:* A H-TDMA was used to measure the water uptake of these chemicals under subsaturated conditions (RH = 85%). Approximately 0.2 g L^{-1} solution was sonicated and aerosolized using a Collison atomizer (Atomizer; TSI 3076). The wet polydisperse aerosols were then exposed to silica gel to dry the particles to RH < 5%. The dry aerosols were then passed through the first Differential Mobility Analyzer (DMA1; TSI 3081) where particles are size selected (40-100 nm) using an electrostatic classifier (TSI 3080). The particles with the right diameter size are then humidified to 85% RH using a nafion humidification line (PermaPure M.H. series). The wet aerosol particles were then scanned using the second DMA (DMA 2) and the size distribution and concentration were measured using a condensation particle counter (CPC; TSI 3776). The growth factor (G_f) for was obtained by dividing the wet diameter size by the dry diameter ($\frac{d_{wet}}{d_{dry}}$). The average growth factor over all diameter sizes were averaged for each chemical and the kappa hygroscopicity parameter was attained.

(b) Supersaturated Measurements: A CCNC (Droplet Measurement Technologies, DMT) was used to measure the water uptake under supersaturated conditions. Approximately 0.2 g L^{-1} solution was sonicated and aerosolized with a Collison Nebulizer (Atomizer; TSI 3076). The aerosolized stream was passed through diffusion dryers to reduce the relative humidity to less than 5%. Post drying, the polydisperse aerosols were then delivered to a Differential Mobility Analyzer (DMA; flow rate: 0.8 L min^{-1}) to produce a monodisperse stream with an equilibrium distribution of charged particles (110). The stream then exits the DMA and splits into two streams. One stream (~0.3 L min^{-1}) flow into the Condensation Particle Counter (CPC; TSI 3776) measuring the particle concentration (*CN*), while the other stream (0.5 L min^{-1}) flow into the CCNC measuring the concentration of activated particles (*CCN*). The activation fractions ($\frac{CCN}{CN}$) and the critical activation diameters (D_d), the diameter size at which 50% of the particle concentration form droplets, were calculated at four different supersaturations (0.46%, 0.67%, 0.86%, and 1.08%) using a Scanning Mobility CCN Analysis (SMCA) (109).

Acid-promoted dissolution experiments and dissolved iron analysis. Three sets of vials, each containing 10 mL AZTD slurries (1 g L^{-1}), were adjusted to pH 1, 3 and 7. The control set had no added organics, and the reaction sets had *o*-aminophenol and *p*-aminophenol with a final concentration of 1 mM. The vials were wrapped in Al-foil and left under continuous stirring for 15 d. Digital photographs were taken as a function of time during the experiment to record the color of the slurries. The pH remained around 1.2±0.1 for the duration of the experiment. Prior to filtration, UV-vis spectra were collected and about 1 mL samples from each set at pH 7 were taken for electron microscopy imaging (see SI for details). Then, the slurries were filtered using nylon membrane filters (0.2 µm pore size, 25 mm diameter, EMD). The filters were left to dry overnight at room temperature before a photograph was taken to show qualitative changes in the optical properties of the dust particles. The filtrate solutions were analyzed for dissolved iron (DFe).

These experiments were conducted to quantify the concentration of total DFe from the simulated dust aging experiments using AZTD at the end of the 15 d. The slurries were filtered using nylon membrane filters (0.2 µm pore size, 25 mm dia., EMD). Dissolved iron concentration in the samples was measured via inductively coupled plasma optical emission spectrometry (ICP-OES, Perkin-Elmer Optima 8000 DV, Perkin Elmer, Woodbridge, Ontario) relative to a standard curve generated using a certified iron stock standard (Perkin Elmer). Samples were introduced to the plasma at a rate of 1 mL min^{-1} via a gemcone concentric nebulizer and a cyclonic spray chamber, and the plasma, nebulizer, and auxiliary argon gas flows were set at 15, 0.7, and 0.5 L min^{-1}, respectively. The RF power was set at 1500 W and the plasma view was set at axial. Final concentrations were blank-corrected. The detection limit of the method (defined as three times the standard deviation of ten consecutive blanks) was calculated to be 0.06 mg L^{-1} and the limit of quantification (defined as ten times the standard deviation of ten consecutive blanks) was calculated to be 0.19 mg L^{-1}.

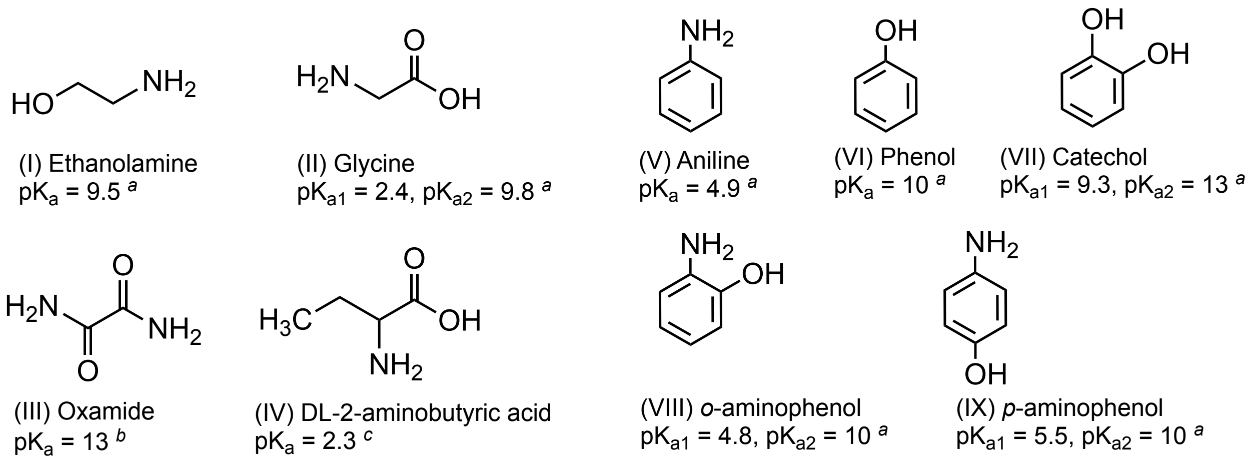

Supplementary Scheme 1. Aliphatic and aromatic amines of atmospheric relevance containing functional groups capable of complexing Fe(III) or undergoing redox reactions. Phenol and catechol are added as a reference compound. Notes: [a] reference[2], [b]*Oxamide (471-46-5)*, <https://www.chemicalbook.com/ProductMSDSDetailCB2495794_EN.htm> [Accessed January 15, 2022], and [c]*DL-2-Aminobutyric acid*, <https://www.chemicalbook.com/ChemicalProductProperty_EN_CB4382245.htm> [Accessed January 15, 2022]

Supplementary Table 1. List of compounds used in this study, their electrochemical oxidation potential and reaction products.

Compound	Oxidation Potential (V)	Cyclic voltammetry experimental conditions	Oxidation reaction	Ref.
Phenol 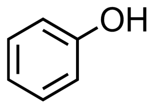	~1<br>(pH 0.2-2)	50 mV s^{-1}<br>vs. Ag/AgCl<br>0.5-25 mM<br>Pt electrode	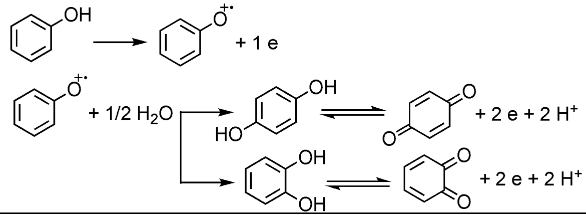	3,4
Catechol 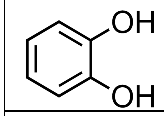	0.43 → 0.2<br>(pH 4→8)	50 mV s^{-1}<br>vs. Ag/AgCl<br>1 mM<br>Pt electrode	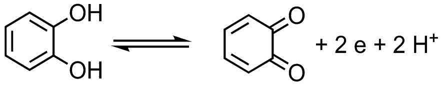	4,5
Aniline 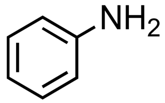	1.26<br>(pH 5)<br><br>1.17<br>(pH 1)(5)	100 mV s^{-1}<br>vs. reversible hydrogen electrode (RHE)<br>1 mM<br>Pt electrode<br><br>50 mV s^{-1}<br>vs.<br>(RHE)<br>10 mM<br>Pt electrode(5)	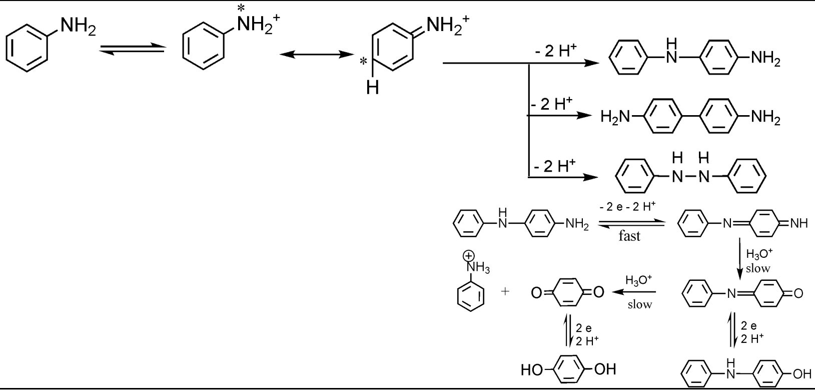	6<br><br>7
o-aminophenol 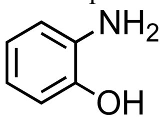	0.6<br>(pH 1)	100 mV s^{-1}<br>vs. Ag/AgCl<br>1 mM<br>Glassy carbon electrode	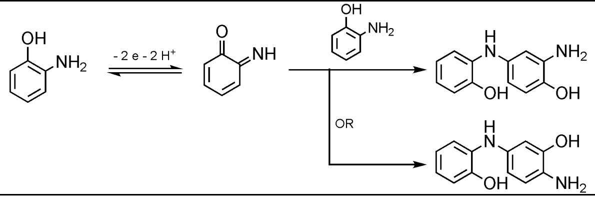	8

p-aminophenol 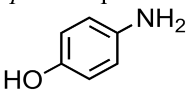	0.4<br>(pH 3)<br><br>0.28<br>(pH 5)	50 mV s^{-1}<br>vs. Ag/AgCl<br>1 mM<br>Glassy carbon electrode	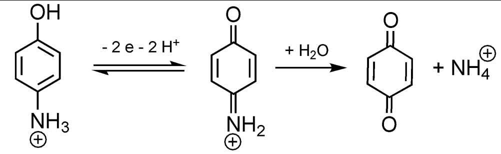	8

Supplementary Table 2. Selected reduction reactions of Fe species and their electrochemical potentials from reference [9]

Reduction reaction	pH range	E (V) range and pH dependency
$Fe(III) + e \rightarrow Fe(II)$	0 – 2.12	0.77
$Fe(OH)^{2+} + H^{+} + e \rightarrow Fe(II) + H_2O$	2.12 – 3.48	0.9 – 0.06 pH<br>(pH 3, E = 0.72 V)
$Fe(OH)_2^{+} + 2H^{+} + e \rightarrow Fe(II) + H_2O$	3.48 – 6.30	1.10 – 0.12 pH<br>(pH 4, E = 0.62 V; pH 5, E = 0.5 V)

Example calculations:

Supplementary equations 1 and 2 represent the complete the redox couple for the net reaction shown in supplementary equation 3:

Oxidation: catechol → ortho-Quinone + 2 e + 2H^{+}, E_{ox1} = 0.4 V (1)

Reduction: $2Fe(OH)^{2+} + 2H^{+} + 2e \rightarrow 2Fe(II) + 2H_2O$, E_{red} = 0.72 V (2)

Net Reaction: catechol + $2Fe(OH)^{2+} \rightarrow o - Quinone + 2Fe(II) + 2H_2O$, $E_{net} = E_{red}\text{-}E_{ox}$ = 0.72-0.4 = 0.32 V (3)

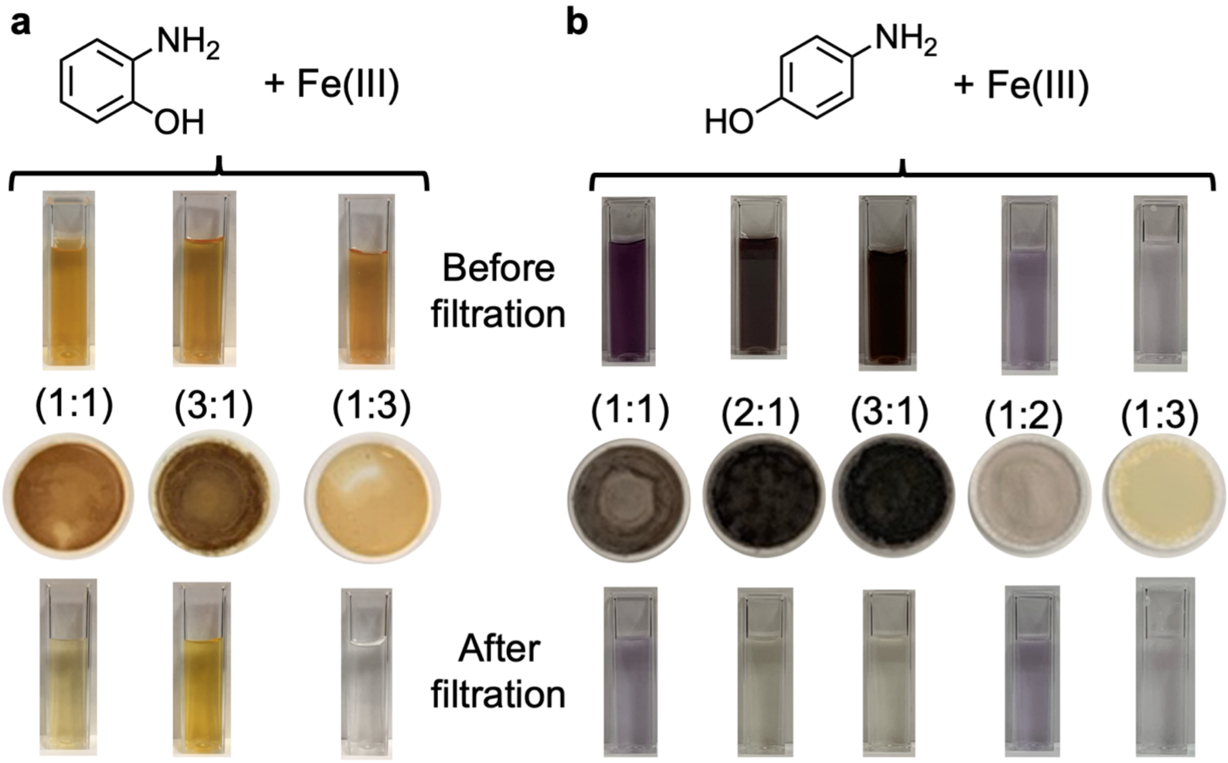

Supplementary Figure 1. Summary of results for the dark aqueous phase reaction of (a) *o*AP and (b) and *p*AP with Fe(III) as a function of the organic to Fe molar ratio showing digital photographs of solutions after 40 min reaction (pH 3-4.5) in 1 M $(NH_4)_2SO_4$ background solution, and dry filters after allowing the reaction to continue overnight (20 hr). For A, [Fe(III)] = 0.5 mM and for B, [Fe(III)] = 1 mM.

Concentration calculations of species in deliquesced aerosols for atmospheric relevance

1) Dissolved iron:

Concentrations in rain, fog, snow, and cloud waters were reported to be location-dependent and varying by over four orders of magnitude, from 0.1 to 10^3 μM.[10] In 20μm cloud droplets, typical concentration of dissolved Fe(III) is 1 μM.

For aerosols,

Typical aerosol liquid water (ALW) content of 6×10^{-8} Lm^{-3} of air

Example iron concentrations in fine particles of 331–1640 ng m^{-3} from across China[11] and the typical lower limit of iron solubility of 5% (wt/wt)

Using the above information, dissolved iron in ALW would range from 5–24 mM according to:

$$\frac{331\,ng}{1\,m^3}\cdot\frac{1\,g\,Fe}{10^9\,g\,Fe}\cdot\frac{1\,mol}{56\,g\,Fe}\cdot\frac{1\,m^3}{6\times10^{-8}L\,water}\cdot 0.05 = 0.005\,M = 5\,mM$$

$$\frac{1640\,ng}{1\,m^3}\cdot\frac{1\,g\,Fe}{10^9\,g\,Fe}\cdot\frac{1\,mol}{56\,g\,Fe}\cdot\frac{1\,m^3}{6\times10^{-8}L\,water}\cdot 0.05 = 0.024\,M = 24\,mM$$

Therefore, the Fe(III) concentration used in our studies that ranged from 0.1-2 mM are atmospherically-relevant for deliquesced aerosols.

2) Dissolved aminophenol concentrations:

Mean *p*-aminophenol (*p*AP) concentration in 30-nm and total suspended particles was estimated to range from 0.09–0.52 ng m^{-3}.[12]

Bulk Solubility, S_{bulk}, of pAP in water[2] equals 15.7 g L^{-1}. Using the density of water, 1 g cm^{-3} (10^3 g L^{-1}), $S_{bulk} = \frac{15.7\,g}{L\,water}\cdot\frac{1\,L\,water}{10^3 g\,water} = 0.0157\frac{g}{g}\cdot 100\% = 1.57\%$

Using the above information, dissolved *p*-aminophenol in ALW would range from 0.2–1.2 μM according to:

$$\frac{0.09\,ng}{1\,m^3}\cdot\frac{1\,g\,pAP}{10^9\,g\,pAP}\cdot\frac{1\,mol}{109.13\,g\,pAP}\cdot\frac{1\,m^3}{6\times10^{-8}L\,water}\cdot 0.0157 = 2\times10^{-7}\,M = 0.2\,\mu M$$

$$\frac{0.52\,ng}{1\,m^3}\cdot\frac{1\,g\,pAP}{10^9\,g\,pAP}\cdot\frac{1\,mol}{109.13\,g\,pAP}\cdot\frac{1\,m^3}{6\times10^{-8}L\,water}\cdot 0.0157 = 12\times10^{-7}\,M = 1.2\,\mu M$$

Using a more recent reference[13] that directly measured aerosol organic nitrogen in ambient aerosol samples at an urban location in Hong Kong, the concentration range was reported to be 0.12-1.41

μg N m^{-3}. Using these numbers and the S_{bulk} for *p*AP above, the dissolved *p*AP in ALW would range from 2.2–26 mM according to:

$$\frac{0.12\ \mu g\ N}{1\ m^3}\cdot\frac{1\ g\ N}{10^6\ \mu g\ N}\cdot\frac{1\ mol\ N}{14\ g\ N}\cdot\frac{1\ mol\ pAP}{1\ mol\ N}\cdot\frac{1\ m^3}{6\times 10^{-8} L\ water}\cdot 0.0157 = 2.2\times 10^{-3}\ M = 2.2\ mM$$

$$\frac{1.41\ \mu g\ N}{1\ m^3}\cdot\frac{1\ g\ N}{10^6\ \mu g\ N}\cdot\frac{1\ mol\ N}{14\ g\ N}\cdot\frac{1\ mol\ pAP}{1\ mol\ N}\cdot\frac{1\ m^3}{6\times 10^{-8} L\ water}\cdot 0.0157 = 26\times 10^{-3}\ M = 26\ mM$$

The bulk solubility of *o*-aminophenol (*o*AP) in water[2] is 19.6 g L^{-1}, 1.2x higher than that of pAP. Therefore, the aminophenol concentrations used in our studies that ranged from 0.03-6 mM are atmospherically-relevant for deliquesced aerosols.

Supplementary Table 3. Mass yields (%)[a] of insoluble BrC from the 2 h reaction time of iron chloride with *o*- and *p*-aminophenol in 101 mL total volume at the specified pH.

**Organic precursor (org)**	**Initial [Org] (mM)**	**Initial [FeCl₃] (mM)**	**pH**	**Background solution**	**% Mass yield**	**Photo of dry filters with particles**
*o*AP	3	1	4.3	0.01 M KCl	13 ± 4	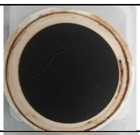
	1	1	3.5		25 ± 6	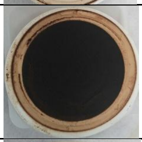
	3	1	4.3	1 M $(NH_4)_2SO_4$	30 ± 7	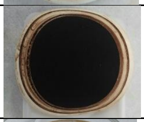
			2.4		9 ± 2	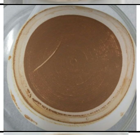
	1	1	4.4		38 ± 10	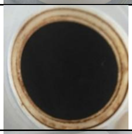
*p*AP	3	1	4.6	0.01 M KCl	23 ± 6	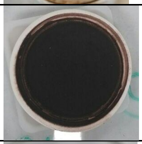
	1	1	3.2		<D.L.>[b]	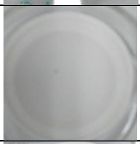
	3	1	2.4	1 M $(NH_4)_2SO_4$	9 ± 2	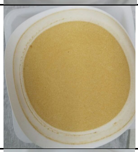
	1	1	4.4		22 ± 6	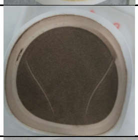
			2.4		<D.L.>[b]	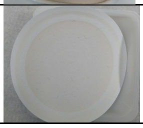

Notes: [a] $Mass\ yield\ (\%) = \frac{mass_{dried\ filter} - mass_{orginal\ filter}}{mass_{organic\ reactant}} \times 100\%$, <D.L.> = below detection limit, 1 mg.

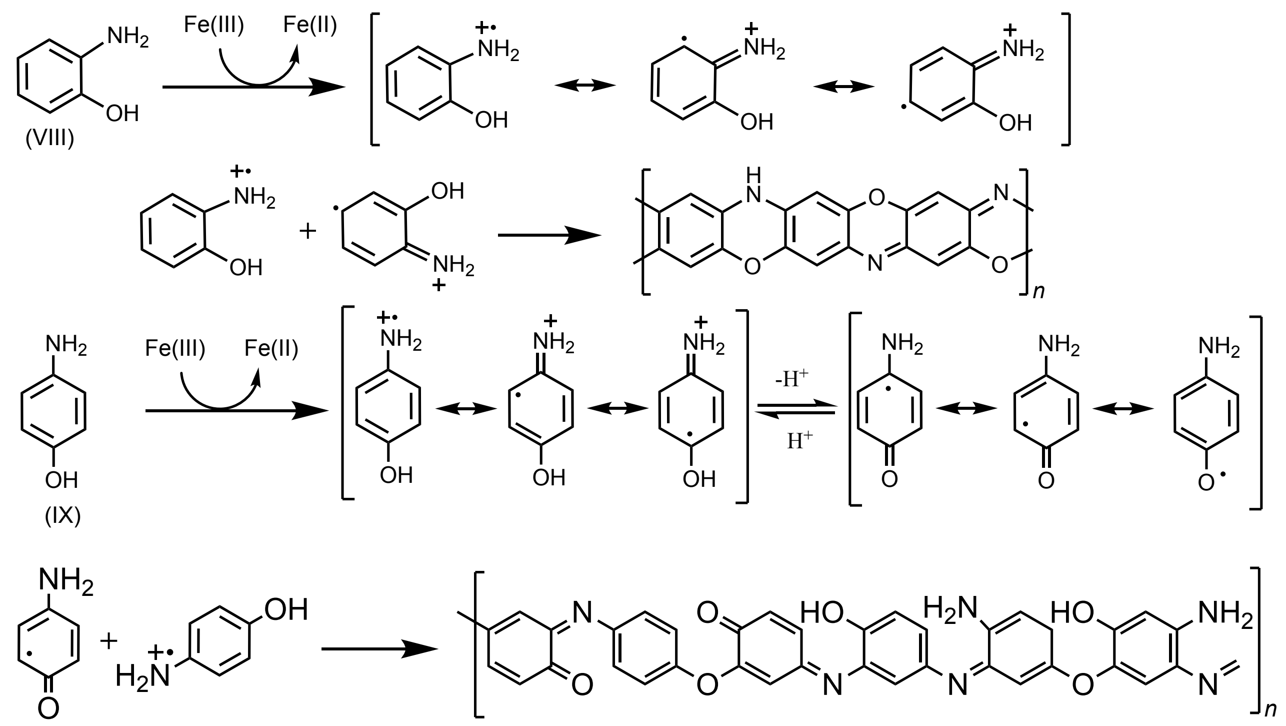

Supplementary Scheme 2. Suggested redox reactions between aminophenols and Fe(III) species that lead to oligomerization. Similar oligomers have been proposed elsewhere.[14]

Supplementary Table 4. Representative Transmission electron microscopy (TEM) coupled with energy dispersive x-ray spectroscopy (EDXS) for elemental analysis of colored particles produced from the reaction of *o*AP and *p*AP with Fe(III). Numbers in parenthesis below the sample number are for electron acceleration voltage and magnification used. As a low mass element, nitrogen is hard to detect with EDXS.

**Particles from reaction of *o*AP with Fe(III)**								
**Sample 1 (200 kV, 50 k)**			**Sample 2 (200 kV, 60 k)**			**Sample 2 (200 kV, 50 k)**		
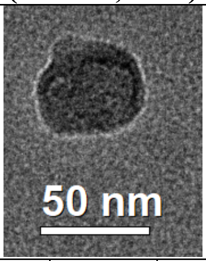			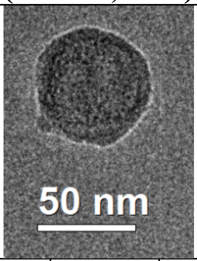			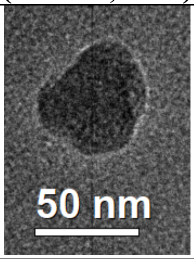		
**Element**	**Wt%**	**Atom%**	**Element**	**wt%**	**Atom%**	**Element**	**wt%**	**Atom%**
**C K**	75	92	**C K**	63	89	**C K**	75	92
**O K**	3.1	2.9	**O K**	2.3	2.4	**O K**	3.8	3.4
**Cl K**	0	0	**Cl K**	0	0	**Cl K**	0.2	0.1
**Fe K**	0.1	0.04	**Fe K**	0.4	0.1	**Fe K**	0.3	0.1
**Particles from reaction of *p*AP with Fe(III)**								
**Sample 1 (200 kV, 40 k)**			**Sample 2 (200 kV, 40 k)**			**Sample 2 (200 kV, 50 k)**		
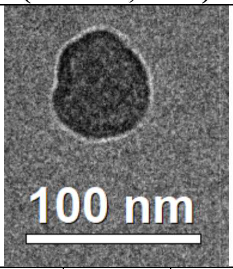			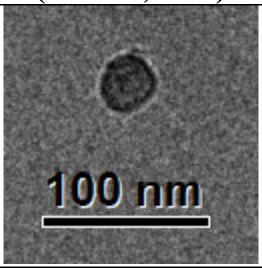			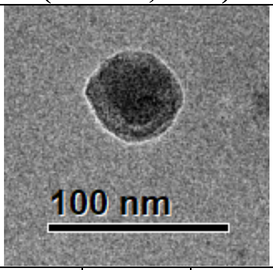		
**Element**	**Wt%**	**Atom%**	**Element**	**wt%**	**Atom%**	**Element**	**wt%**	**Atom%**
**C K**	35	73	**C K**	56	87	**C K**	44	78
**O K**	1.7	2.6	**O K**	2.1	2.4	**O K**	2.6	3.5
**Cl K**	0	0	**Cl K**	0	0	**Cl K**	0.4	0.3
**Fe K**	0.6	0.3	**Fe K**	0.5	0.2	**Fe K**	1.0	0.4

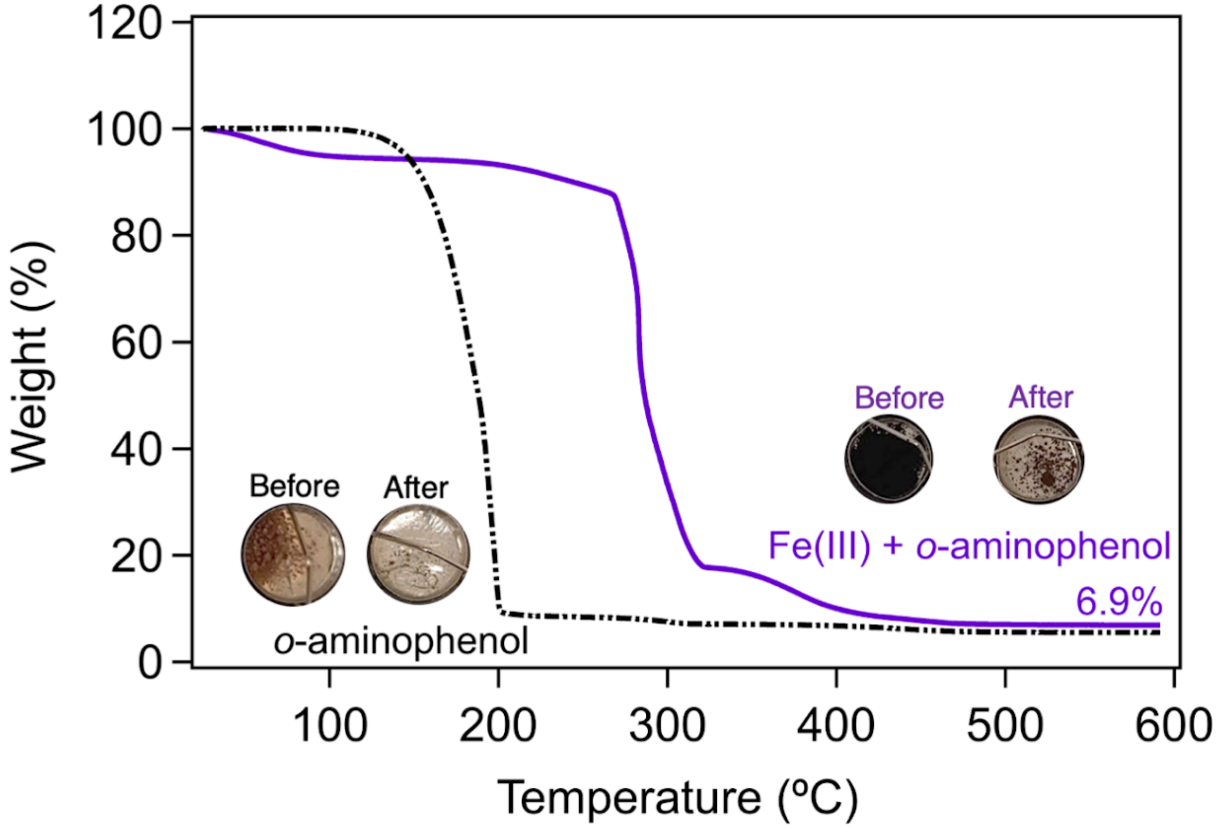

Supplementary Figure 2. TGA curves in air for the organic reactants and insoluble particles forming from the reaction of *o*-aminophenol (*o*AP) with Fe(III) under acidic conditions.

Supplementary Table 5: Analysis of the TGA data (n.a. = not available)

**Reaction for thermal decomposition**	**% mass residual, (c1)**	**% Fe calculated from c1, (c2)**	**Calculated molar weight (g mol⁻¹) from c2 (c3)**
Fe-*o*AP ($FeC_xO_yH_zN_k$)→ 0.5x Fe_2O_3 + gases	6.9	4.8	1159

Notes: Values in columns c2 and c3 were calculated according to supplementary equations 4 and 5:

$$\%Fe\ (y) = \frac{x\ g\ Fe_2O_3}{100\ g\ sample} \cdot \frac{2\ mol\ Fe}{1\ mol\ Fe_2O_3} \cdot \frac{1\ mol\ Fe_2O_3}{160\ g\ Fe_2O_3} \cdot \frac{56\ g\ Fe}{1\ mol\ Fe} \cdot 100\% \quad (4)$$

$$Molar\ weight\ (w) = \frac{100\ g\ sample}{y\ g\ Fe} \cdot \frac{56\ g\ Fe}{1\ mol} \quad (5)$$

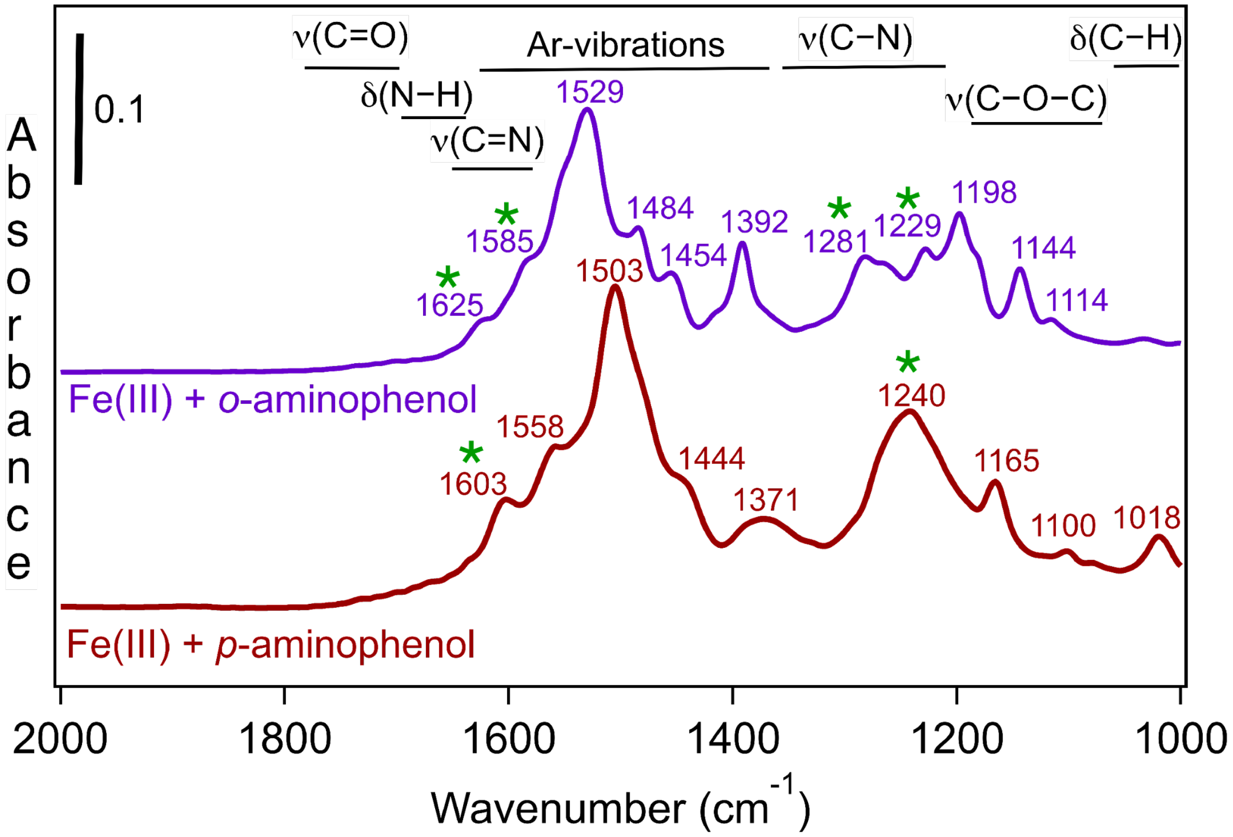

Supplementary Figure 3. ATR-FTIR absorbance spectra of thin films of the solid products formed from the reaction of Fe(III) with *o*-aminophenol (*o*AP, top) and *p*-aminophenol (*p*AP, bottom) under acidic conditions. The thin films were deposited on a ZnSe ATR crystal from a water/ethanol slurry followed by drying overnight. The '*' highlights spectral features for nitrogen-containing bonds.

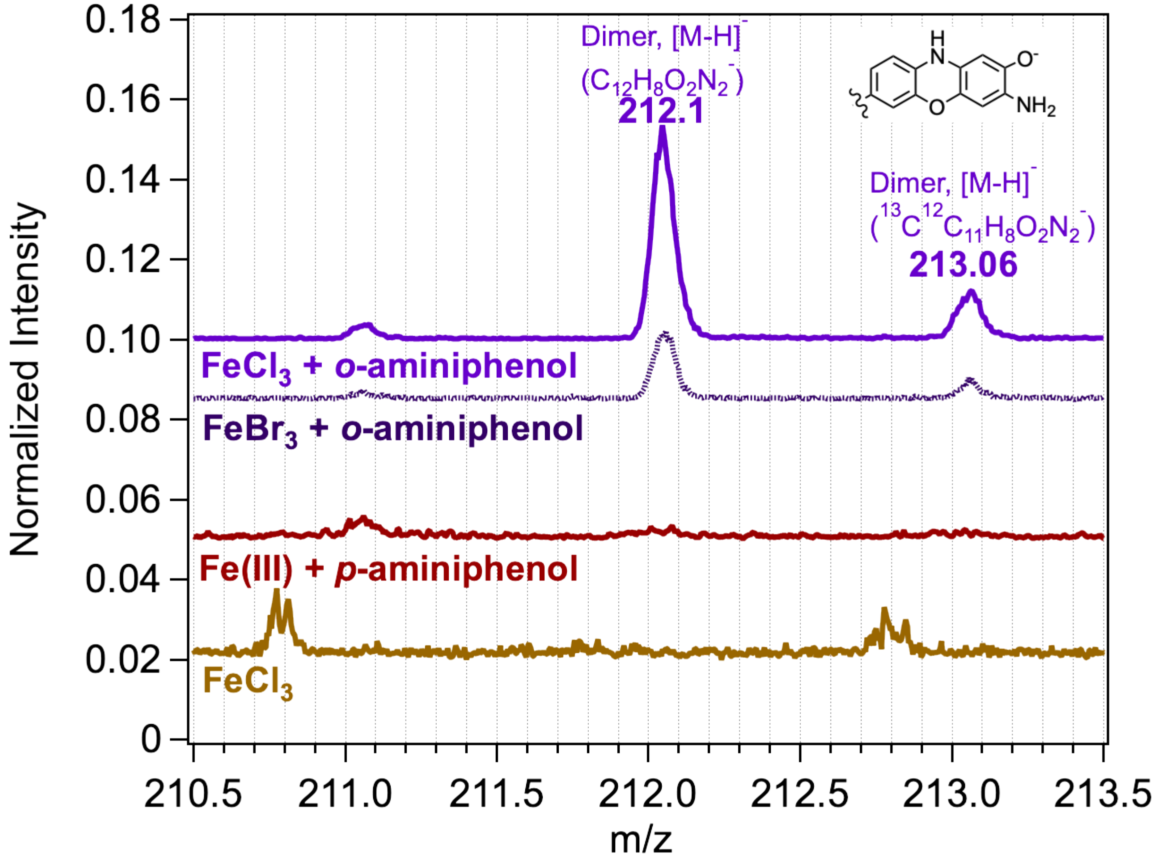

Supplementary Figure 4. Negative mode MALDI spectra in the m/z 210.5 – 213.5 of matrix-free solid thin films formed from the reaction of Fe(III) with *o*-aminophenol (*o*AP, top) and *p*-aminophenol (*p*AP, middle) under acidic conditions. The spectrum using reactant $FeCl_3$ (bottom) is shown as a reference with very weak features likely arising from impurities.

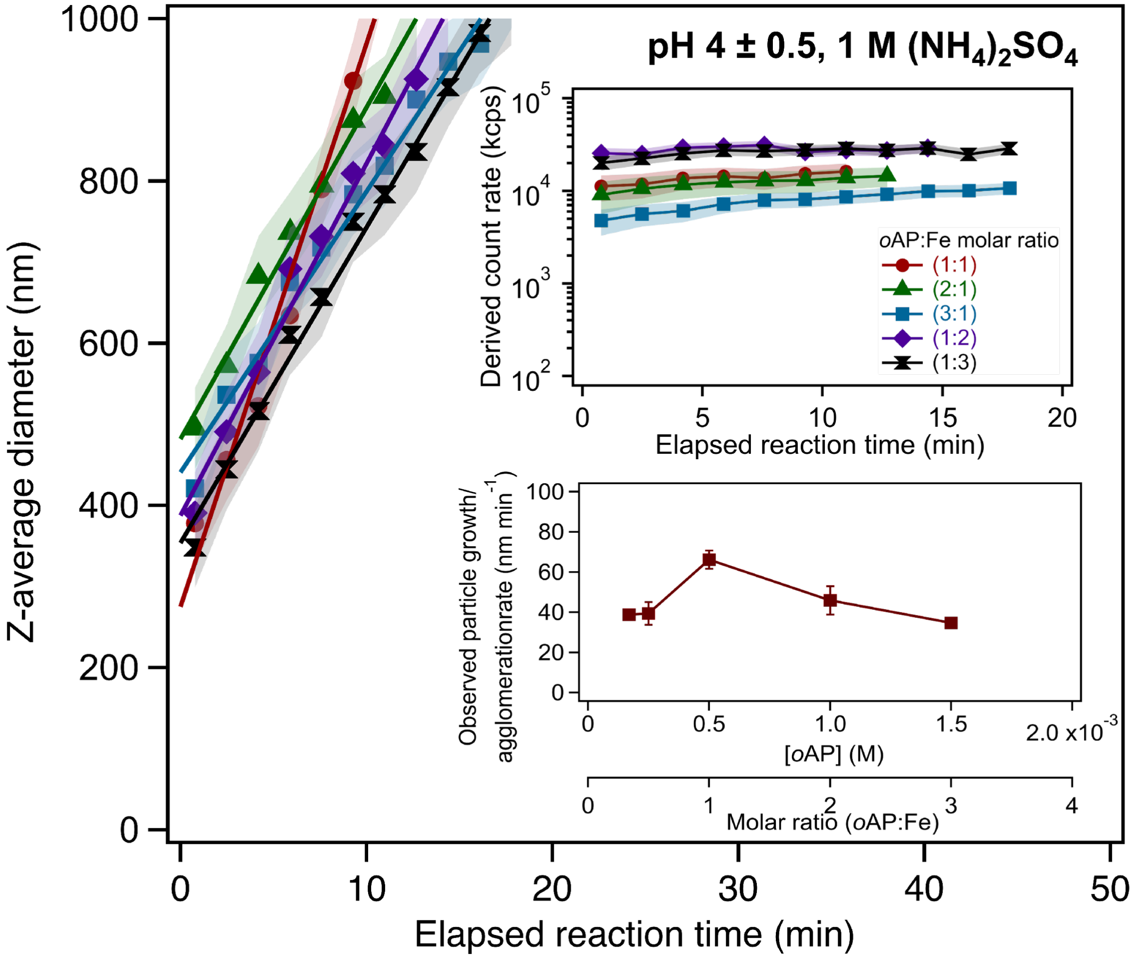

Supplementary Figure 5. Effect of ionic strength on particle size from the reaction of *o*AP with Fe(III). DLS measurements of the average particle size of particles as a function of time (±1σ = 30%). Lines through the data correspond to linear least squares fitting. (Inset) Derived count rate, proportional to the scattering signal, from the particles forming in solution (upper), and observed rate of particle growth or aggregation as a function of [*o*AP] and molar ratio of *o*AP:Fe from the DLS data with error bars represent ±1σ (lower). [Fe(III)] = $5 \cdot 10^{-4}$ M.

a

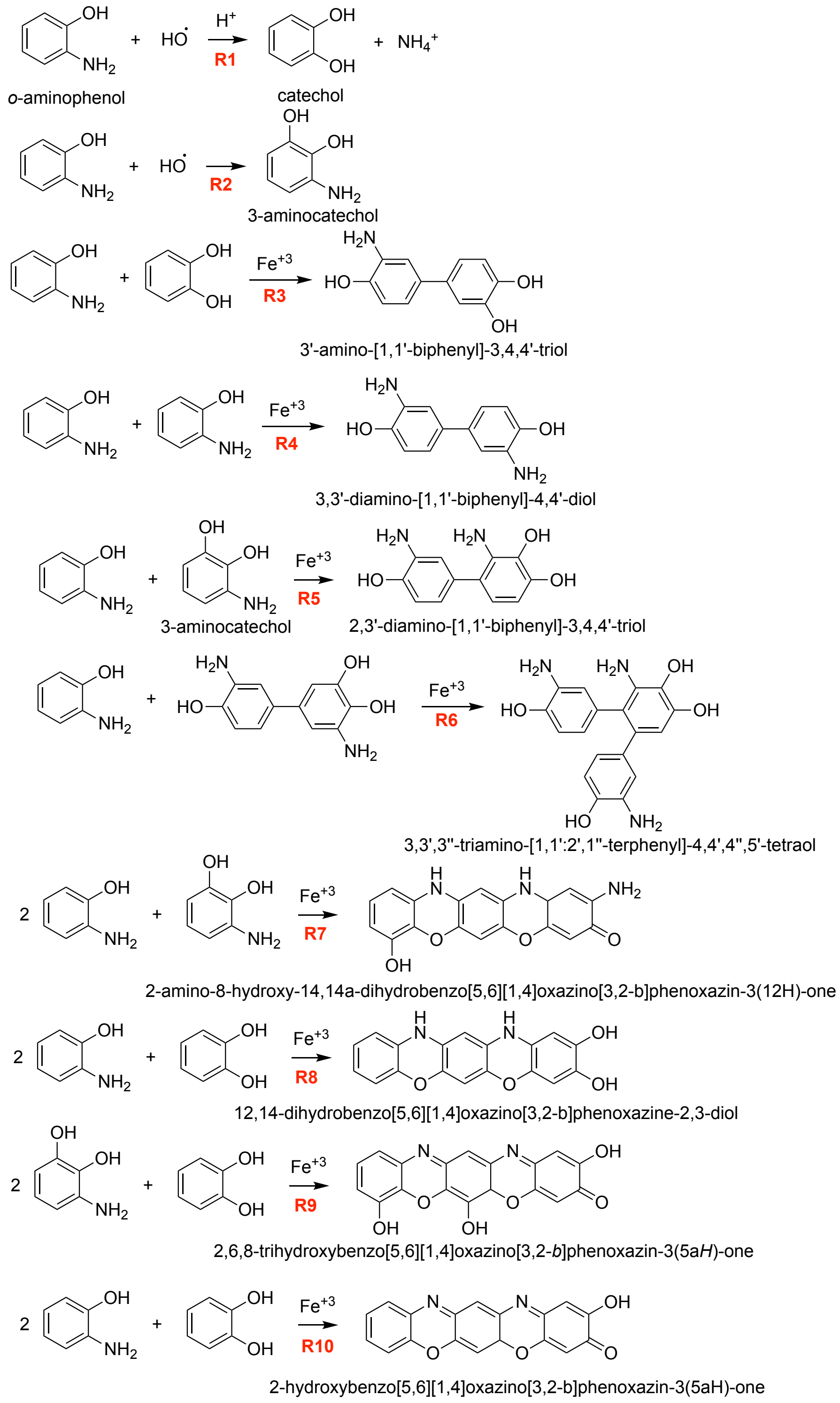

Supplementary Scheme 3. Suggested formation reactions for the structures of dimers and trimers identified in the UPLC-MS experiments from the reaction of (a) *o*-aminophenol and (b) *p*-aminophenol with Fe(III).

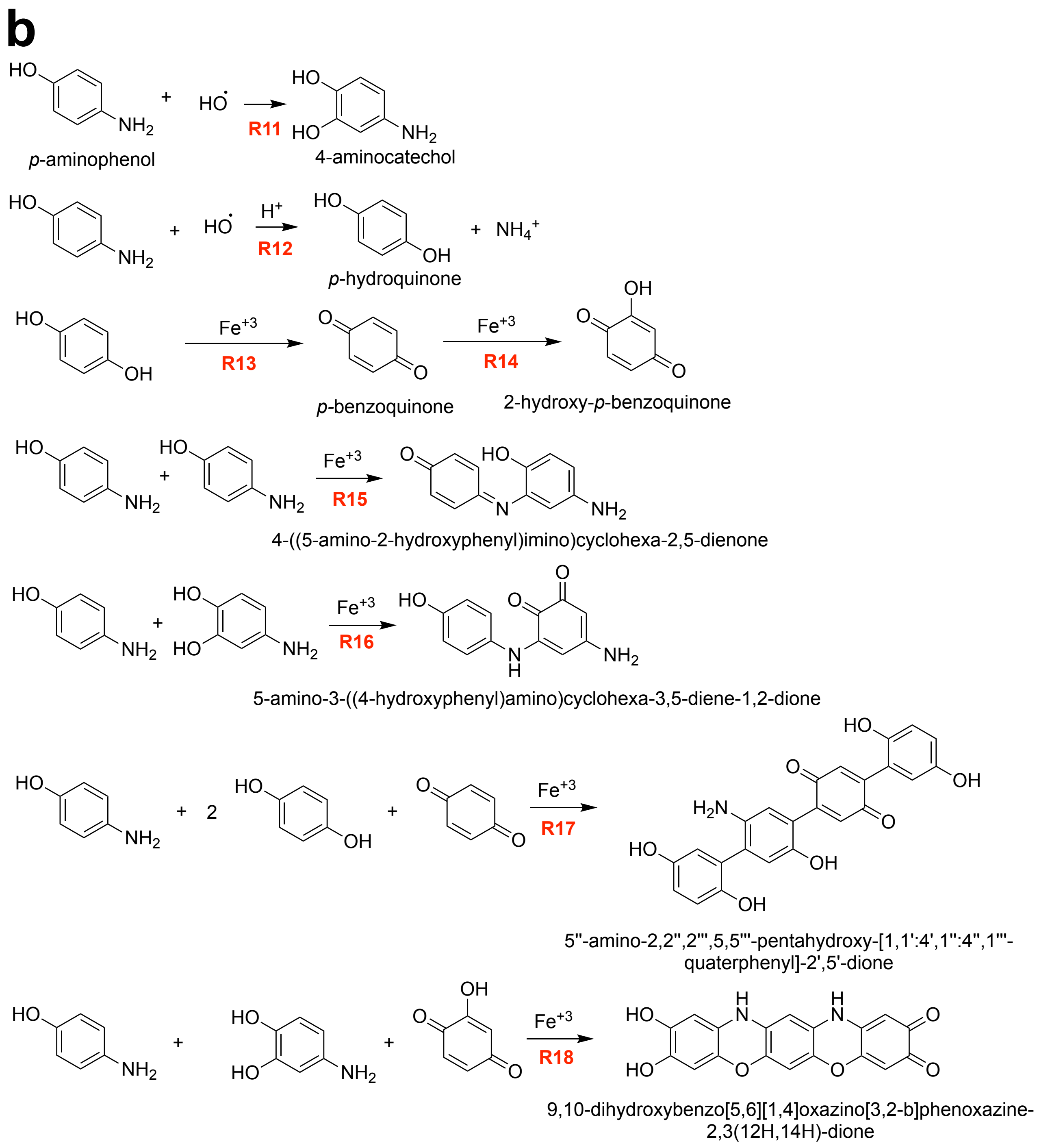

..to be continued, Supplementary Scheme 3: Suggested formation reactions for the structures of dimers and trimers identified in the UHPLC-MS experiments from the reaction of (a) *o*-aminophenol and (b) *p*-aminophenol with Fe(III).

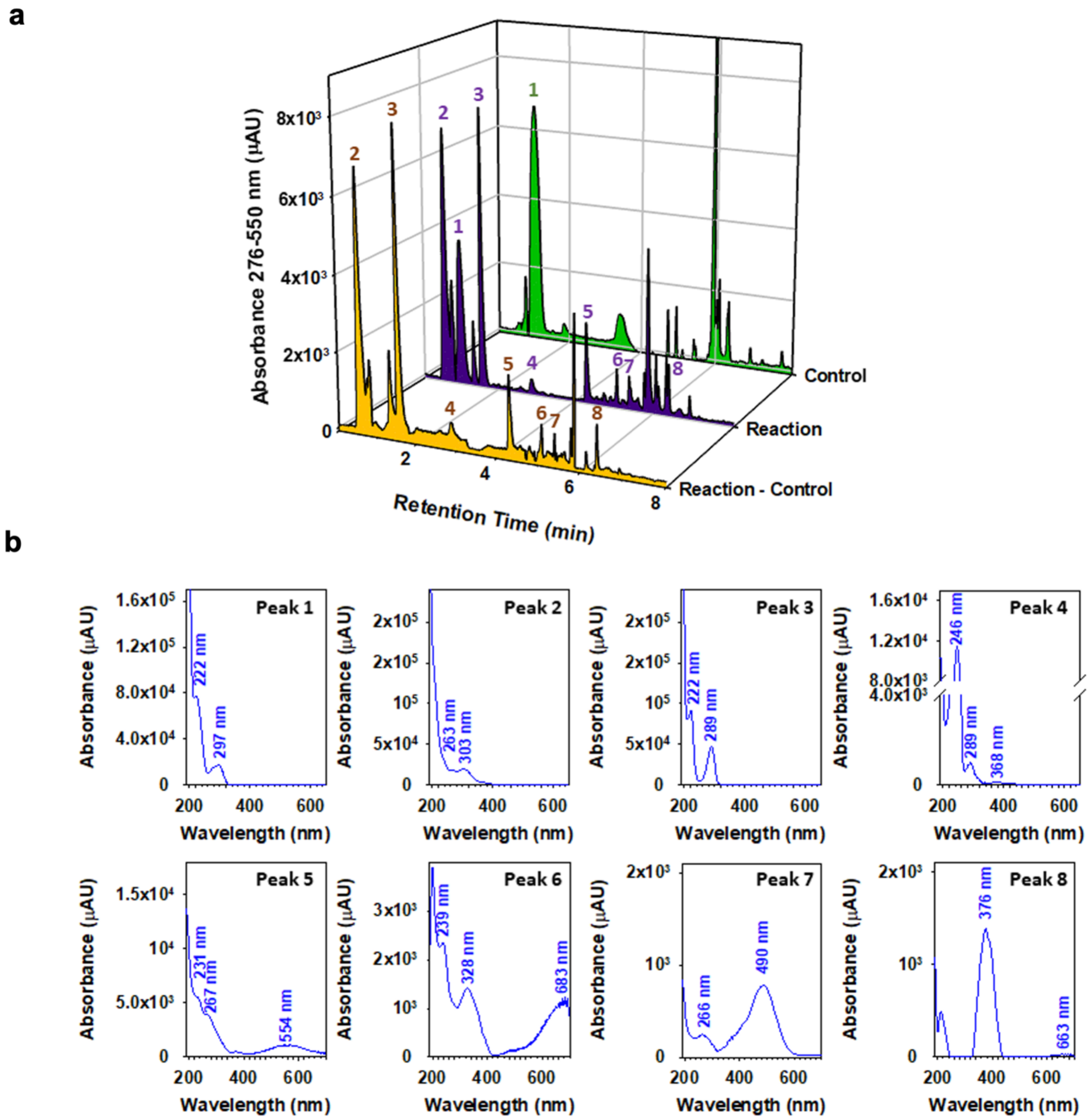

Supplementary Figure 6. (a) UV-visible (276-550 nm) chromatogram with additional ESI/MS(+) detection for the filtrates following the overnight reaction of (purple) *p*AP (labeled as peak 1) with Fe(III) in a 3:1 ratio, (green) a control in the absence of Fe(III), and (yellow) the remaining positive signal after subtraction of the control to the reaction. Labeled product peaks 2-8 are discussed in the text. Detailed reaction conditions and concentrations are the same listed in Fig. 1. (b) Extracted UV–visible spectra of peaks 1 through 8 in the chromatograms of Fig. 5.

For comparison of the retention times of 8 chromatographic peaks, standards of 4-nitrophenol, catechol, hydroquinone, *p*-benzoquinone, and 4,4'-dihydroxybiphenyl were analyzed.

Supplementary Figure 6a shows in green a control without Fe(III), and the result from subtracting the chromatogram of the control to that of the reaction in yellow. The elution of *p*AP (peak 1 in Supplementary Figure 6a) at t_r = 0.96 min provides a well-resolved peak to register the UV-visible spectrum reported in Supplementary Figure6b with λ_{max} = 222 and 297 nm. The ion for peak 1 in Supplementary Figure 6a is observed at m/z^+ 110.14 (M = C_6H_7ON) for $M+H^+$, and at m/z^+ 151.10 for the adduct with acetonitrile and proton. Peak 2 (t_r = 0.56 min) with λ_{max} = 263 and 303 nm, forms an adduct with NH_4^+ at m/z^+ 143.14 for a smaller parent peak at m/z^+ 126.13, corresponding to the formation of 4-aminocatechol (or an isomer) as displayed in reaction R11 of Scheme S3B..

Peak 3 (t_r = 1.54 min) in Supplementary Figure 6a with λ_{max} = 222 and 289 nm, and m/z^+ 241.14 corresponds to (as confirmed with a standard) an ammonium adduct of *p*-hydroquinone (formed via reaction R12 of Scheme S3B) with acetonitrile and four water molecules. The elution of peak 4 for *p*-benzoquinone at t_r = 2.88 min with λ_{max} = 246, 289 and 368 nm, as depicted in reaction R13 (Scheme 3B), was also confirmed with a standard to explain the observed ion at m/z^+ 126.11 for the ammonium adduct. Reaction R14 in Scheme 3B is used to display the formation of a hydroxylated product from *p*-benzoquinone (e.g., 2-hydroxy-*p*-benzoquinone) that can contribute to the monomers of trimers eluting al later retention times. Peak 5 (t_r = 4.30 min) displayed absorption maxima at 231, 267 and 554 nm forming an ion at m/z^+ 215.24 of general formula $C_{12}H_{11}O_2N_2^+$ for the proton adduct of a *p*AP dimer. The quinone functionality in peak 5 of Supplementary Figure 6b can be explained by a structure such as that of 4-((5-amino-2-hydroxyphenyl)imino)cyclohexa-2,5-dienone (Reaction R15 in Scheme S3B). Peak 6 in Supplementary Figure 6b elutes at t_r = 5.09 min with λ_{max} = 239, 328 and 683 nm, and m/z^+ 313.26. Peak 6 is interpreted as a proton adduct for a dimer between *p*AP with 4-aminocatechol with two molecules of acetonitrile, which was oxidized to quinone as in the structure of 5-amino-3-((4-hydroxyphenyl)amino)cyclohexa-3,5-diene-1,2-

dione (reaction R16 in Scheme S3B). Peak 7 (t_r = 5.49) with λ_{max} = 266 and 490 nm and m/z$^+$ 449.28 is described by an ammonium adduct of a tetramer of *p*AP + 2 × hydroquinone + *p*-quinone molecules that loses 8 H atoms during coupling reactions. Such an ion, $C_{24}H_{21}O_7N_2^+$, and absorption features could be explained by the formation of 5''-amino-2,2'',2''',5,5'''-pentahydroxy-[1,1':4',1'':4'',1'''-quaterphenyl]-2',5'-dione (Reaction R17 in Scheme S3B). Finally, peak 8 (t_r = 6.40) with λ_{max} = 376 and 498 nm, and m/z$^+$ 351.14 represents a proton adduct for a trimer among *p*AP + 4-aminocatechol + 2-hydroxy-*p*-benzoquinone such as 9,10-dihydroxybenzo[5,6][1,4]oxazino[3,2-b]phenoxazine-2,3(12H,14H)-dione (Reaction R18 in Scheme S3B).

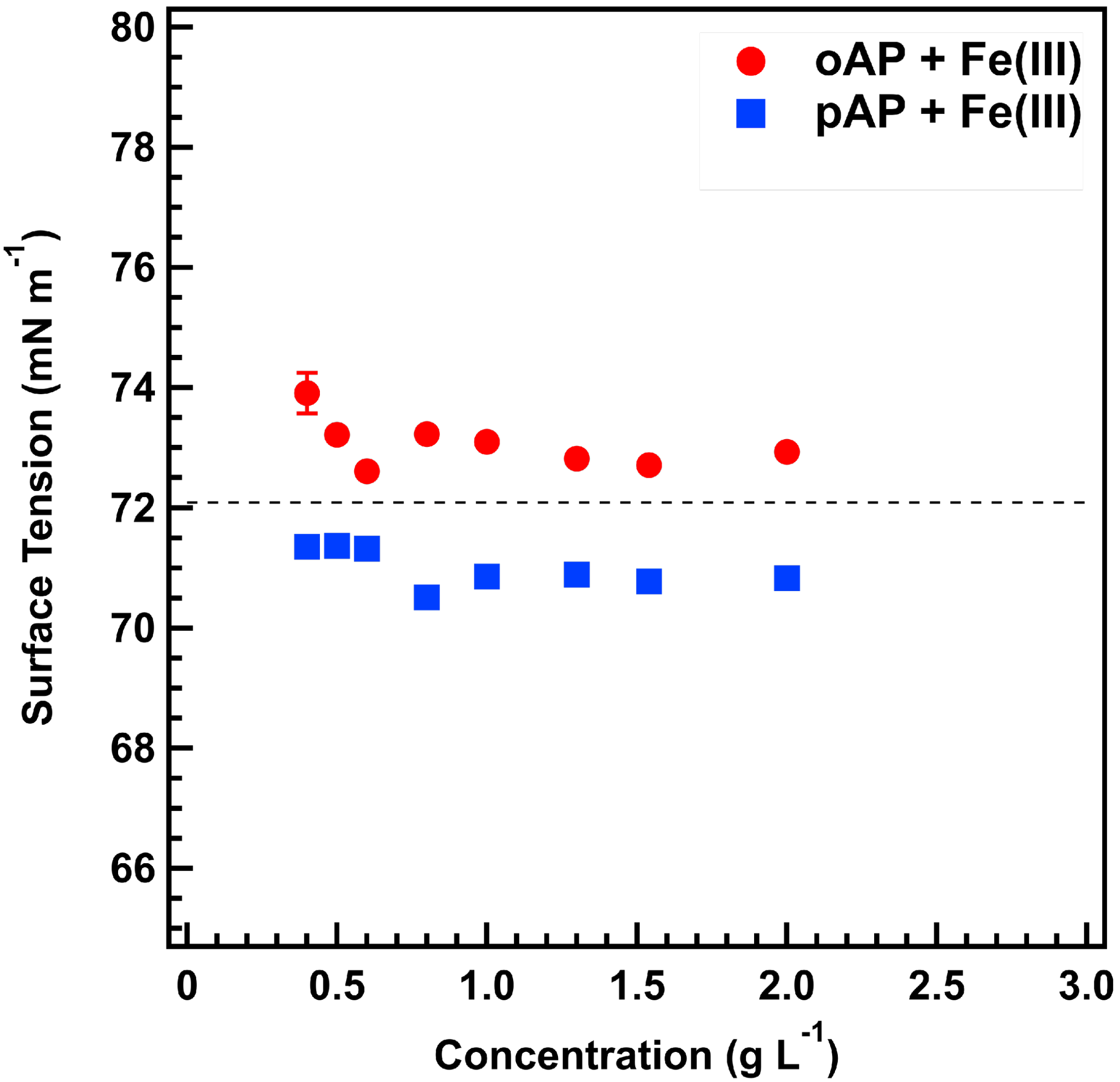

Supplementary Figure 7. Surface tension measurements for insoluble oligomers from the reaction of Fe(III) with *o*AP (red circles) and *p*AP (blue circles). Horizontal line represents the surface tension of pure water, 72.27 mN m^{-1}. Room temperature: 21.8 °C. Error bars represent standard deviation with similar length to the marker thickness.

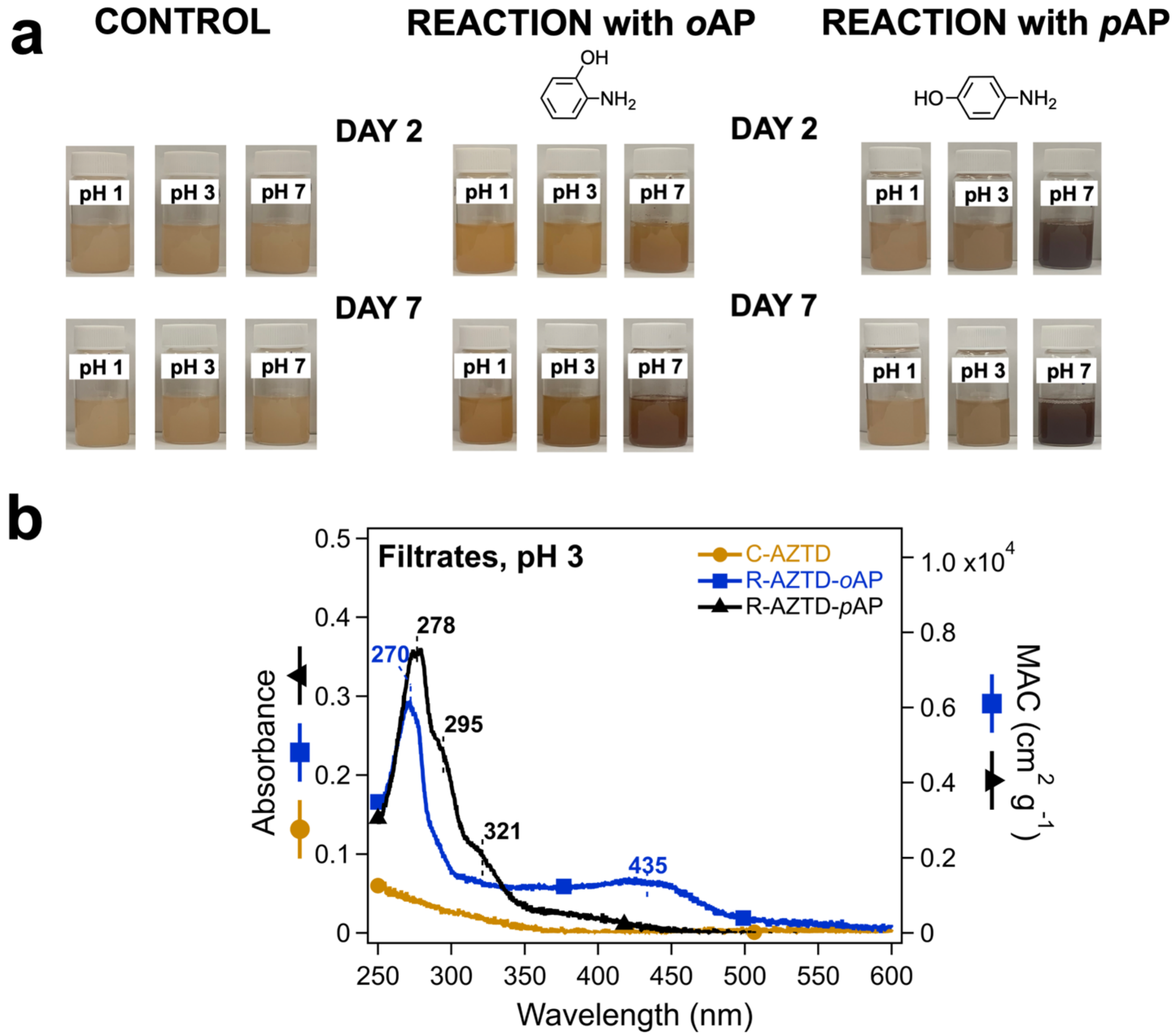

Supplementary Figure 8. (a) Digital photographs of control (left), reaction with *o*-aminophenol (*o*AP, middle), and *p*-aminophenol (*p*AP, right) vials containing AZTD as a function of pH after 2 and 7 d of simulated atmospheric aging time. The pH values shown in the label of each vial refers to the starting pH of the slurry. (b) Absorbance spectra (left) as a function of wavelength for filtrates following the 15 d reaction of AZTD at pH 3 in the absence of organics (C-AZTD) and reacted with *o*AP (R-AZTD-*o*AP) and *p*AP (R-AZTD-*p*AP). MAC spectra (right) are shown for filtrates from reacted samples using the initial mass concentration of *o*AP and *p*AP of 1 mM (1.1×10^{-4} g mL^{-1}).

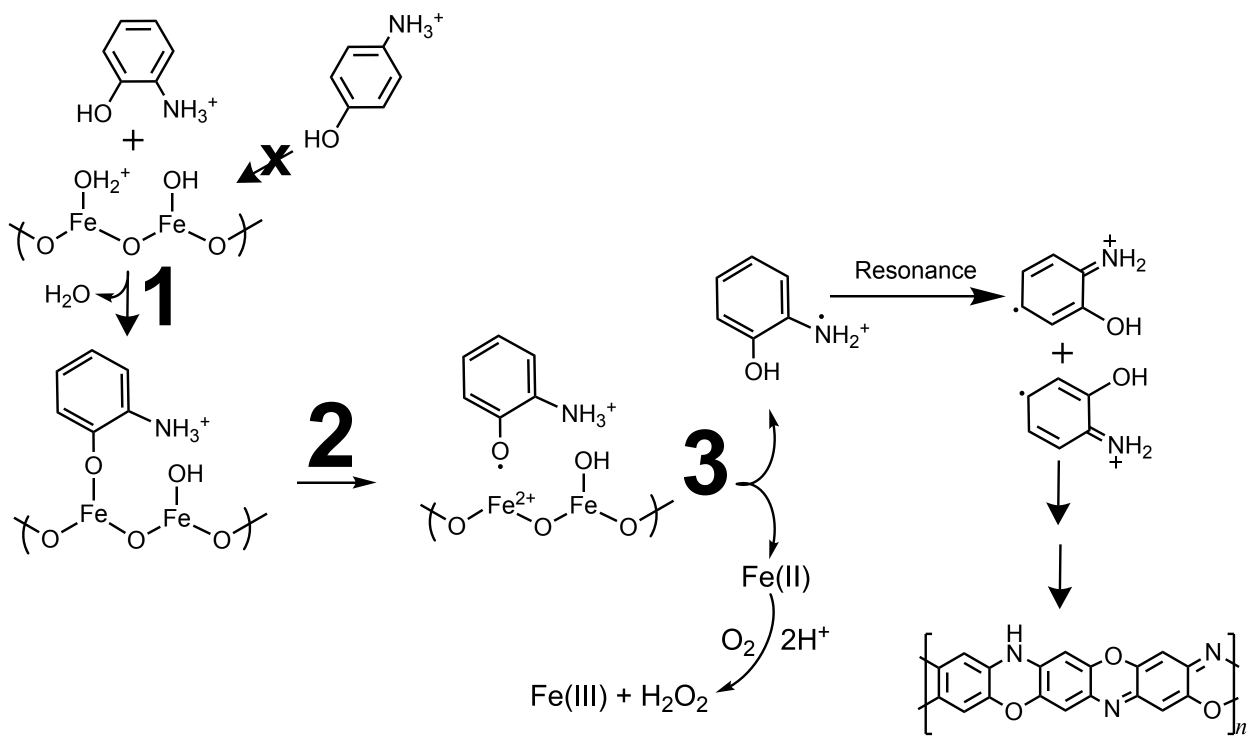

Supplementary Scheme 4. Suggested mechanism for surface complexation and reaction of *o*AP and *p*AP with iron (oxyhydr)oxide sites on AZTD at pH 1.

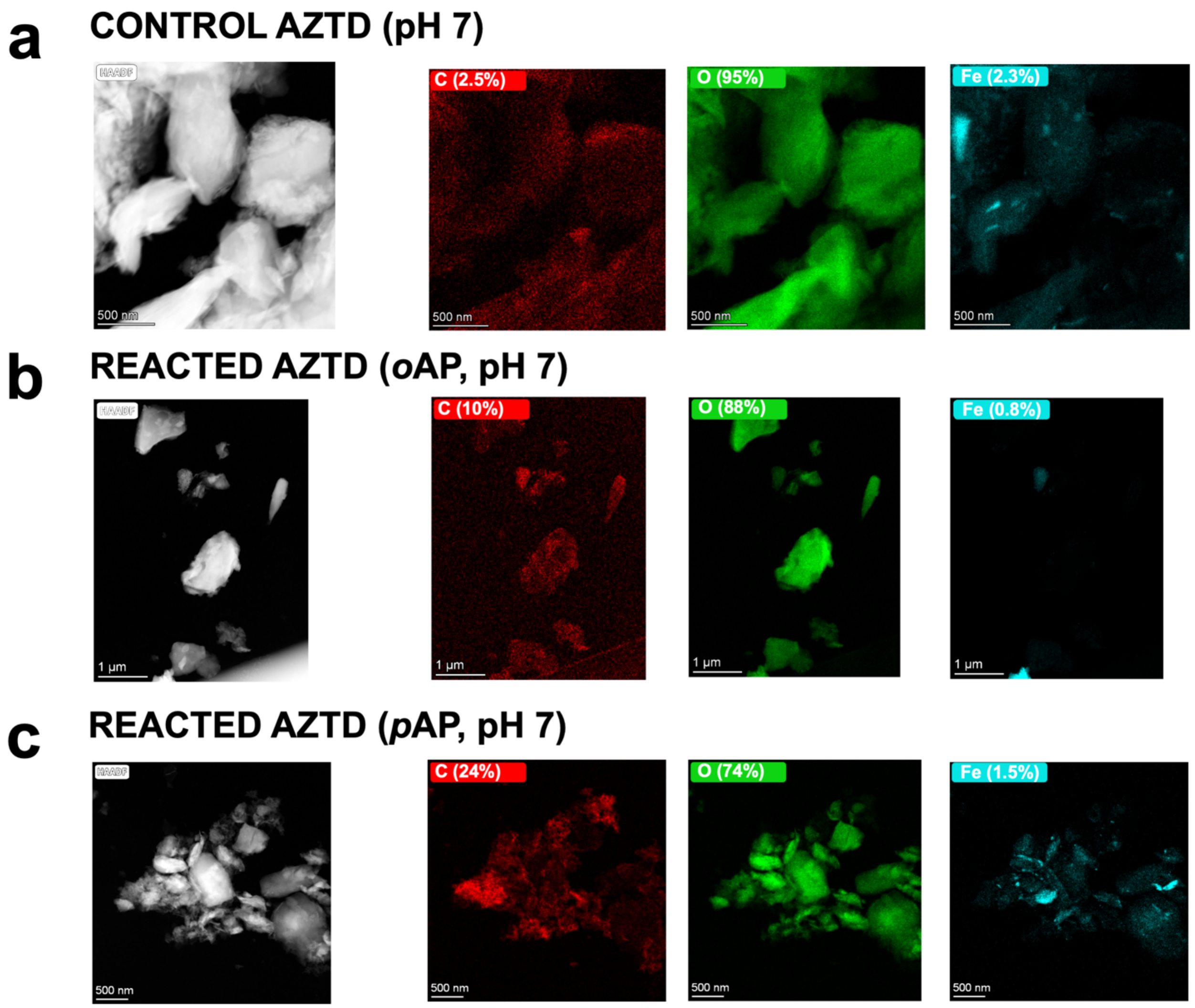

Supplementary Figure 9. Scanning transmission electron (STEM) microscopy images with elemental mapping of AZTD following 15 d of simulated atmospheric aging time at pH 7 for the (a) control (no added organics), (b) reaction with *o*-aminophenol (*o*AP), and (c) reaction with *p*-aminophenol (*p*AP). The percentages listed are % atomic fraction.

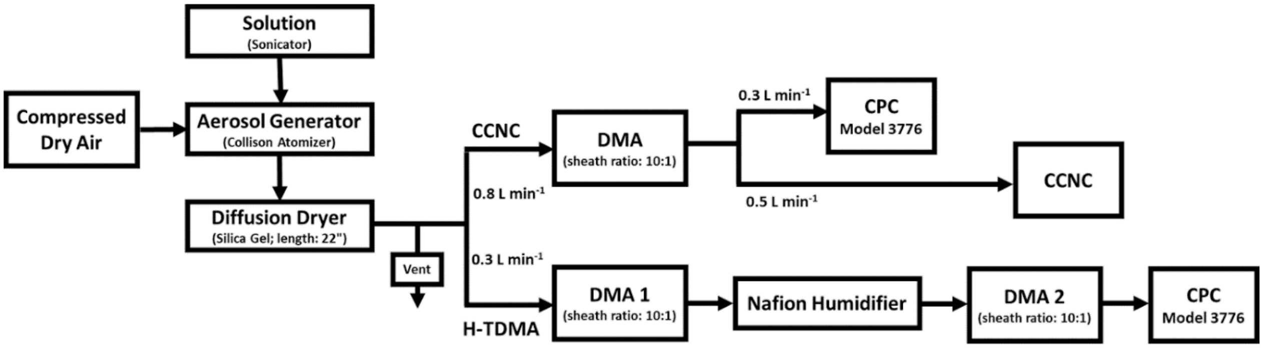

Supplementary Figure 10. Experimental setup for the CCNC and H-TDMA measurements.

Supplementary References:

1 Al-Abadleh, H. A., Rana, M. S., Mohammed, W. & Guzman, M. I. Dark Iron-Catalyzed Reactions in Acidic and Viscous Aerosol Systems Efficiently Form Secondary Brown Carbon. *Environ. Sci. Technol.* **55**, 209-219 (2021).

2 Lide, D. R. *CRC Handbook of Chemistry and Physics*. Vol. 93 (Taylor & Francis, 2012-2013).

3 Xavier, J. L. N., Ortega, E., Ferreira, J. Z., Bernardes, A. M. & Perez-Herranz, V. An electrochemical Study of Phenol Oxidation in Acidic Medium. *Int. J. Electrochem. Sci.* **6**, 622-636 (2011).

4 Chiorcea-Paquim, A. M., Enache, T. A., Gil, E. D. & Oliveira-Brett, A. M. Natural phenolic antioxidants electrochemistry: Towards a new food science methodology. *Comrr. Rev. Food Sci. F.* **19**, 1680-1726 (2020).

5 Kasa, T. & Solomon, T. Cyclic Voltammetric and Electrochemical Simulation Studies on the Electro-Oxidation of Catechol in the Presence of 4, 4-bipyridine. *Am. J. Phys. Chem.* **5**, 45-55 (2016).

6 Cases, F., Huerta, F., Garces, P., Morallon, E. & Vazquez, J. L. Voltammetric and in situ FTIRS study of the electrochemical oxidation of aniline from aqueous solutions buffered at pH 5. *J. Electroanaly. Chem.* **501**, 186-192 (2001).

7 Yang, H. & Bard, A. J. The application of fast scan cyclic voltammetry. Mechanistic study of the initial stage of electropolymerization of aniline in aqueous solutions. *J. Electroanal. Chem.* **339**, 423-449 (1992).

8 Beiginejad, H., Nematollahi, D. & Varmaghani, F. Electrochemical Oxidation of Some Aminophenols in Various pHs. *J. Electrochem. Soc.* **160**, H41-H46 (2013).

9 Channei, D. *et al.* Aqueous and Surface Chemistries of Photocatalytic Fe-Doped CeO_2 Nanoparticles. *Catalysts* **7**, 1-23 (2017).

10 Deguillaume, L. *et al.* Transition Metals in Atmospheric Liquid Phases: Sources, Reactivity, and Sensitive Parameters. *Chem. Rev.* **105**, 3388-3431 (2005).

11 Gen, M., Zhang, R., Li, Y. & Chan, C. K. Multiphase Photochemistry of Iron-Chloride Containing Particles as a Source of Aqueous Chlorine Radicals and Its Effect on Sulfate Production. *Environ. Sci. Technol.* **54**, 9862-9871 (2020).

12 Ruiz-Limenez, J. *et al.* Aliphatic and aromatic amines in atmospheric aerosol particles : Comparison of three ionization techniques in liquid chromatography masss spectrometry and method development. *Talanta* **97**, 55-62 (2012).

13 Yu, X. *et al.* Simultaneous Determination of Aerosol Inorganic and Organic Nitrogen by Thermal Evolution and Chemiluminescence Detection. *Environ. Sci. Technol.* **55**, 11579-11589 (2021).

14 Thenmozhi, G., Arockiasamy, P. & Santhi, J. Isomers of Poly Aminophenol: Chemical Synthesis, Characterization, and Its Corrosion Protection Aspect on Mild Steel in 1M HCl. *Int. J. Electrochem.* **2014**, 1-12 (2014).
